# The expression of father-daughter bond behaviors influences adult partner attachment in titi monkeys

**DOI:** 10.1038/s41598-025-31143-6

**Published:** 2025-12-16

**Authors:** Lynea R. Witczak, Allison R. Lau, Brad A. Hobson, Sara M. Freeman, Pauline B. Zablocki-Thomas, Madison Dufek, Emilio Ferrer, Abhijit J. Chaudhari, Karen L. Bales

**Affiliations:** 1 Department of Psychology, University of California, Davis, Davis, CA USA; 2 California National Primate Research Center, University of California, Davis, Davis, CA USA; 3 Graduate Program in Animal Behavior, University of California, Davis, Davis, CA USA; 4 Center for Molecular and Genomic Imaging, University of California, Davis, Davis, CA USA; 5 Department of Biology, Utah State University, Logan, UT USA; 6 Department of Radiology, University of California, Davis, Davis, CA USA; 7 Department of Neurobiology, Physiology, and Behavior, University of California, Davis, Davis, CA USA; 8 Department of Biology, 100 Campus Drive, Elon, NC 95616 USA

**Keywords:** Neuroimaging, Preference test, Pair bond, Filial bond, Social salience network, Stress buffering, Psychology, Neuroscience, Social behaviour, Animal behaviour, Behavioural methods, Imaging, Behavioural ecology

## Abstract

Coppery titi monkeys (*Plecturocebus cupreus*) are socially monogamous monkeys that display strong pair bonds similar to human romantic attachments, preceded by infant attachment to their fathers. To understand how father-daughter bonds impact adult relationship dynamics, we established a novel method for quantifying expression of bond-related behaviors. We assessed behavioral and neural correlates of preference, stress buffering, and separation distress to identify how females’ current and former attachment figures impact female attachment. Whereas all females (*n* = 9) shifted to preferring their partner over father six-months post-pairing, females that exhibited higher expression of juvenile parent preference maintained a relationship with their father six-months post-pairing, as evidenced by higher-than-expected father proximity. Higher expression of juvenile measures of proximity following a brief separation predicted slightly increased partner proximity in adulthood. Neural activity patterns in brain regions assessed pre- and post-pairing showed high similarity in glucose metabolism, despite overall activity being lower post-pairing. While there was some inconsistency in results, higher expression of juvenile proximity following a separation was associated with enhanced reduction in activity within social bonding brain regions (social salience network, periaqueductal gray, cerebellum), suggesting a potential stress buffering benefit via reduced threat-related brain activation, like that seen in high-quality human relationships. These findings advance current knowledge of how early relationships may shape adult bond-related behavior and neural activity.

**Supplementary Information:**

The online version contains supplementary material available at 10.1038/s41598-025-31143-6.

## Introduction

Social interactions regulate behavioral, psychological, and physiological processes^1^, with supportive relationships linked to healthier habits^2^, effective stress buffering^1^, and overall improved health, potentially contributing to increased longevity^3^. A lack of social connections is associated with adverse health outcomes^4^, including higher risks of coronary heart disease and stroke^5^. Social bonds are enduring, selective relationships maintained by both physiological and behavioral mechanisms^6,7^, as described by attachment theory^8,9^, and these bonds manifest through synchronized dyadic behaviors^10–12^ in various forms including parent-offspring bonds, pair bonds, and friendships. Pair bonds are long-lasting relationships between two unrelated adults^6^, characterized by a preference for partner proximity^13^, joint and cooperative aggression toward intruders^14,15^, separation distress^16^, and mutual stress buffering^17,18^. Mating supports pair bonds^7,19^ but is not essential for pair bond formation^6^. Pair bonds are often the most important social bond in an adult’s life, and understanding what factors impact variation in pair bonds may provide insights into ensuring healthier, longer lives.

Titi monkeys (*Plecturocebus spp.*) are socially monogamous South American monkeys that live in small family groups^20^ and form pair bonds^21^, making them an excellent model for social bond research with human health applications. In titi monkeys, fathers are the primary attachment figures for infants^22^. Fathers spend more time carrying infants than mothers, which leads to a clear infant preference for paternal contact over maternal interaction^22^. Titi monkey infants exhibit clear signs of distress—such as vocalizations, locomotion, and elevated cortisol levels—when separated from their fathers, but not when the father is present^22,23^. This strong paternal attachment endures into later development, as daughters continue to show prolonged stress responses during separations that subside only upon reunion with their fathers, indicating long-lasting filial bonds^24^. As adults, titi monkeys form exclusive pair bonds that mimic the early filial attachment^25^; separation from an adult mate (also referred to as a partner, or a pair mate) triggers significant stress responses that are alleviated only upon reunion, unlike separations from other family members^22,26^. Although juveniles primarily rely on their fathers and adults on their partners, there is notable individual variation in attachment behaviors^27–30^, suggesting that differences in early infant–father relationships might influence later adult bonding patterns.

Research in humans shows that early attachment relationships, especially during adolescence, strongly shape initial romantic bonds^31–33^. For example, adolescents who experienced nurturant-involved parenting later developed warm, supportive relationships with their romatic partners^34^. Another study found college students who reported avoidant attachment relationships with their parents had lower scores on the Perceived Relationship Quality Scale as young adults in romatic relationships^35^. Titi monkeys provide experimental control as translational models to study the first transition to adult attachment. In the wild, titi monkeys gradually shift away from family groups, increasing interactions with unfamiliar conspecifics and naturally emigrating around 3–4 years of age^36–39^. The effect of father–daughter bonds on transitioning from natal group living to forming long-term pair bonds remains unclear, but evidence indicates that stronger early paternal attachments may predict greater partner affiliation^40,41^ and lower anxiety-like behaviors in adulthood^41^, likely via underlying neurobiological mechanisms. It is possible that early relationships may shape other aspects of adult bonds, including preference for maintaining proximity to and distress upon involuntary separation from the attachment figure.

Whereas prairie voles have long served as the model for understanding the neural substrates of pair bonding^42,43^, recent neuroimaging studies in titi monkeys (including both sexes) have identified key brain regions—such as the ventral pallidum, nucleus accumbens, and lateral septum—that are integral to monogamous bonds^44–47^. In prairie voles, coordinated activity among oxytocin, vasopressin, and dopamine in regions like the nucleus accumbens and ventral pallidum is critical for establishing and maintaining pair bonds^7^, with receptor dynamics shifting to reinforce partner preference and exclude unfamiliar individuals^48^. Short-term separations in titi monkeys lead to decreased neural activity in regions linked to reward and stress regulation (e.g., ventral pallidum, lateral septum, paraventricular nucleus of the hypothalamus, periaqueductal gray) along with increases in oxytocin and cortisol, suggesting an interactive role between the HPA axis and attachment-related neural circuits during relationship distress^49^. The behavioral parallels between daughters’ attachments to their fathers and adult pair bonds imply that similar neurobiological mechanisms may underlie both types of bonds^19^, supporting the hypothesis that adult pair bonds may have evolved from parental attachment systems^50–53^. The amygdala appears to play a more prominent role in adult romantic attachment than in infant attachment; its deactivation in humans reduces social inhibition^54^ while lesioning in non-human primates reduces social inhibition and facilitates affiliation^55^, indicating that its suppression may help initiate pair bonding in adults.

Whereas each brain region may play a unique part in supporting social interactions, it has been hypothesized that social information is encoded in a dynamic manner across networks of brain regions. As a result, behavior may be more strongly linked to patterns of neural activity across a network, rather than in any one given brain region^56^. One network that is hypothesized to be important for selective social attachments, like pair bonds and parent-offspring bonds, is the social salience network. This neural network has been defined and tested in pair bonding prairie voles and includes the nucleus accumbens, medial amygdala, basolateral amygdala, paraventricular nucleus of the hypothalamus, and ventral tegmental area^57^. The relevance of the social salience network has also been tested in juvenile and adult titi monkeys^45,58^, with additional brain regions included—such as the lateral septum and basal ganglia—based on their hypothesized importance for titi monkey pair bonds^21^. The social salience network is likely connected to the periaqueductal gray and cerebellum due to their expression of oxytocin or vasopressin receptors^59,60^. The periaqueductal gray has been implicated in pair bonds in titi monkeys^49^, humans^61^, and pair bonding rodents^62^ whereas the cerebellum has been associated with partner separation distress in titi monkeys^49^.

Our understanding of the neural correlates of infant attachment—and how they compare to adult romantic attachment within the same individual—is limited, especially because most of our initial research on the neurobiology of titi monkey attachment focused on adult males. Therefore, it is critical to identify brain regions activated in parallel paradigms when titi monkey females interact with their fathers versus their partners, as the expression of father-daughter bond-related behaviors may significantly influence neural activity in regions associated with social bonding. To measure expression of father-daughter bond-related behaviors, we used the same methods as those used in previous studies from our lab^29,30^. Briefly, using data from historical scan samples and experimental manipulations conducted when females were infants and juveniles, we quantified 12 measures that fell into one of three categories of behaviors important for social bonds: distress upon separation from the attachment figure^16^, preference for maintaining close social proximity to the attachment figure^13^, and affiliative partner-directed behaviors^6^. For the present study, we defined higher expression of father-daughter bond-related behaviors as greater expression of these three categories of behaviors relative to the sample mean.

Our primary objectives were to determine how expression of father-daughter bond-related behaviors affects both the behavioral correlates of proximity maintenance and the neural correlates of separation distress and stress buffering. To address these objectives, we conducted two separate pre- and post-pairing experiments (Supplementary Fig. S1): (1) three preference tests comparing time spent near the father versus the partner one-week pre-pairing, one-week post-pairing, and six-months post-pairing, and (2) a neuroimaging study consisting of four [^18^F]-fluorodeoxyglucose Positron Emission Tomography ([^18^F]FDG PET) scans investigating brain glucose metabolism and plasma cortisol during temporary (30-minute) separations from current attachment figures compared to metabolism while with their current attachment figure (father while in the natal group one-month pre-pairing, partner six-months post-pairing). The three preference tests allowed us to assess changes in who the female prefers to maintain proximity to over time, whereas the four PET scans allowed us to compare changes in neural indicators of separation distress and stress buffering pre- and post-pairing. We specifically wanted to assess transitions from father-daughter bonds to pair bonds to better understand the behavioral and neurological changes that may exist in the wild at the time when females emigrate from the natal group to form a pair bond while remaining near their parents’ territory; as well as to add to our general framework of knowledge about neural changes that occur when transitioning from a developmental attachment relationship to an adult attachment relationship. To our knowledge, this is the first study to directly assess simultaneous preference between the father and partner for female titi monkeys as well as the first to directly compare the stress buffering abilities of the primary attachment figures (father pre-pairing and partner post-pairing). We formulated several specific predictions:

### Experiment 1 predictions

1. Over time, females will shift from preferring to spend more time with their father^63^ to spending more time with their partner^64^.
2. Higher expression of infant and juvenile father-daughter bond-related behaviors will positively correlate with time in proximity to the current attachment figure (father during one-week pre-pairing and one-week post-pairing tests, partner during six-month post-pairing test).

### Experiment 2 predictions

1. At six months post-pairing, there will be increased glucose metabolism in the social salience network (amygdala, hypothalamus, lateral septum, nucleus accumbens, ventral pallidum, and ventral tegmental area), periaqueductal gray, cerebellum, and whole brain^46,49^; however, we predict decreased glucose metabolism during social separation conditions in these brain regions important for pair bonding^49^ (Supplementary Fig. S2). We also predict elevated plasma cortisol in response to separation conditions.
2. Higher expression of father-daughter bond-related behaviors will result in further reduced glucose metabolism in all brain regions of interest as a result of the buffering abilities of social bonds, which can reduce threat-related neural activity in those in high-quality relationships^65,66^.

## Results

### Experiment 1: prediction 1

We first assessed females’ preference for spending time near their father compared to their partner during a series of three preference tests (one-week pre-pairing, one-week post-pairing, six-months post-pairing). We predicted that females would shift towards preferring to spend more time near their partner from the first (one-week pre-pairing) to the third (six-month post-pairing) preference tests. Whereas the amount of time females spent near their father across the three tests remained fairly stable, females increased the amount of time they spent near their partner from the first to third test and exhibited a preference for the partner over the father at the six-month post-pairing timepoint.

**Fig. 1:**
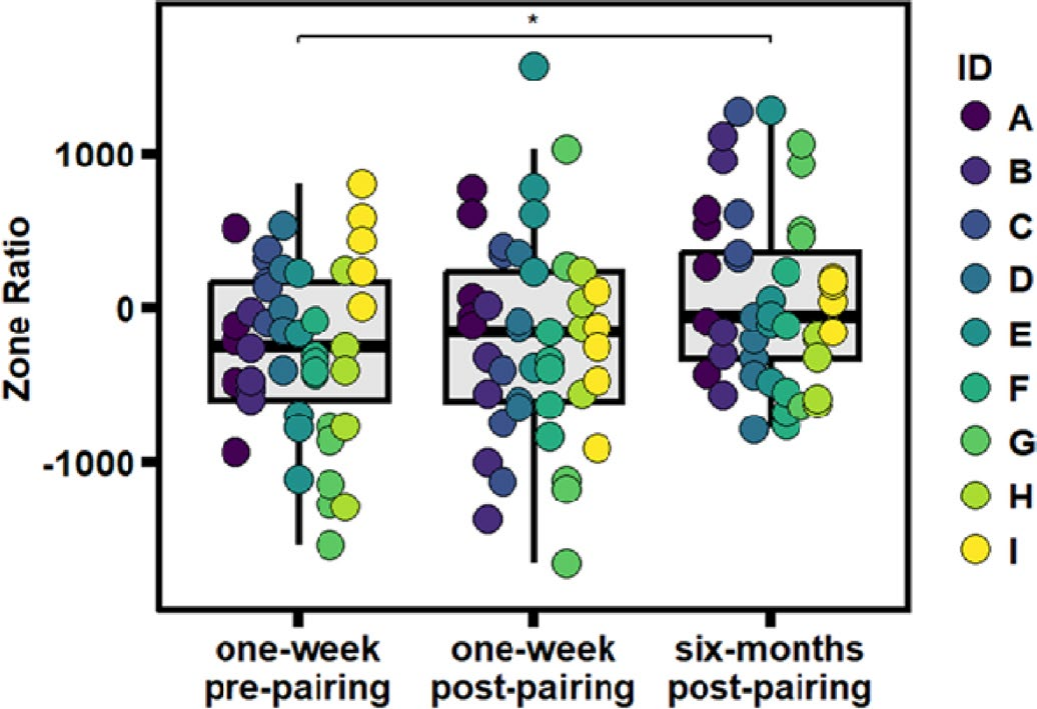
The main effect of *Test Number* on Zone Ratio score. During the one-week pre-pairing and one-week post-pairing tests, females spent more time in proximity to their fathers (more negative scores indicate a greater preference for the father over the partner); however, at six-months post-pairing, females spent slightly more time in proximity to their partners (more positive scores indicate a greater preference for the partner over the father). This preference for the partner over the father was significantly different when comparing the one-week pre-pairing results to the six-months post-pairing results. However, preference for the father over the partner was not statistically significantly different between the one-week post-pairing and six-month post-pairing time points, representing a slight shift in preference for the partner one-week post-pairing, despite no statistically significant difference between Zone Ratio scores for the one-week pre- and post- pairing tests. Points on the graph are colored based on subject identity. Significant differences for pairwise comparisons between tests indicated as: * < 0.05; ** < 0.01; *** < 0.001.

We first calculated a *Zone Ratio* score to assess which stimulus animal females spent more time near as an indication of preference (positive values represented more time in the partner’s preference zone whereas negative values represented more time in the father’s preference zone). As predicted, during the one-week pre-pairing and one-week post-pairing tests, females spent more time in proximity to their fathers (Mean = −270.41, SD = 551.01, and Mean = −210.90, SD = 655.02, respectively; Fig. 1). At six-months post-pairing, females shifted to preferring the partner over the father (Mean = 58.47, SD = 562.49; Fig. 1), but this difference was less pronounced than females’ preferences for spending time in proximity to their partners over strangers in other studies^13,30,64^. Based on our best-fitting model for *Zone Ratio* (R^2^ = 0.131), females spent significantly more time in proximity to their partners during the six-months post-pairing test compared to the one-week pre-pairing test (β = 328.90, SE = 119.92, *t =* 2.74, *p =*.019, *f*^*2*^ = 0.067; Fig. 1). Whereas there was no difference in *Zone Ratio* scores between the one-week pre-pairing and one-week post-pairing tests (β = 59.51, SE = 119.92, *t =* 0.496, *p =*.873, *f*^*2*^ = 0.067; Fig. 1, Supplementary Table S1a) as a result of females showing a slight increase in preference for the partner one-week post-pairing, there also was no significant difference between the one-week post-pairing and six-months post-pairing tests (β = 269.40, SE = 119.92, *t =* 2.25, *p =*.068, *f*^*2*^ = 0.067; Fig. 1).

The *Zone Ratio* results were further supported by the results examining time females spent separately in their partner’s and father’s preference zones. Based on the best-fitting *Partner Zone* model (R^2^ = 0.258, Supplementary Table S1b), there was no significant difference in time spent in the partner’s zone between the one-week pre- and one-week post-pairing tests (β = 0.521, SE = 1.36, *t =* 0.384, *p =*.922); however, females spent significantly more time in their partner’s preference zone during the six-month post-pairing test compared to the one-week pre-pairing test (β = 5.20, SE = 1.36, *t =* 3.83, *p <*.001) and the one-week post-pairing test (β = 4.67, SE = 1.36, *t =* 3.44, *p =*.002, *f*^*2*^ = 0.333; Supplementary Fig. S3). Interestingly, the best-fitting *Father Zone* model (R^2^ = 0.109, Supplementary Table S1c) suggested females did not significantly change in the amount of time they spent in proximity to their fathers across all three tests (Supplementary Fig. S4). In contrast to the preference zone results, when we examined time spent touching the partner’s and father’s grates, females did not significantly change the amount of time they spent touching their partner’s grate (Supplementary Fig. S5, Supplementary Table S1d) or their father’s grate (Supplementary Fig. S6, Supplementary Table S1e) across all three tests.

### Experiment 1: prediction 2

In addition to examining overall patterns of preference and time in proximity to the father and partner, we examined how measures of infant and juvenile bond-related behaviors were associated with proximity behaviors across the three adult preference tests. Of the 12 infant and juvenile bond-related measures assessed from historical scan samples and experiments, five measures from three experiments significantly explained variability in adult behavior: (1) percentage of time juveniles spent in their parents’ preference zone during a juvenile preference test (*Juvenile Parent Preference*), (2) percentage of time juveniles spent in proximity, contact, or tail-twining with their fathers following a 30-minute separation test (*Juvenile Proximity*), (3) percentage of times juveniles chose their parents over strangers following a brief separation during a catch and release test (*Juvenile Parent Choice*), (4) percentage of time infants spent in proximity to the father during an infant open field test (*IOF Proximity*), and (5) percent change in vocalizations when separated from the father compared to when tested with the father during infant open field testing (*IOF Vocalizations*).

We predicted that females exhibiting a greater expression of bond-related behaviors as juveniles would demonstrate a further increased preference for their current attachment figure across tests. However, our prediction was generally not supported. Females that preferred to spend more time near their parents during juvenile parent preference testing (*Juvenile Parent Preference*) also preferred to spend more time near their fathers and less time near their partners during the present adult testing. Interestingly, if females spent more time in proximity to their fathers as juveniles following a brief separation (*Juvenile Proximity* and *Juvenile Choice*), then they spent more time in proximity to their partners during the three adult preference tests; however, the effect size for these interactions were small so interpretation requires caution. These different measures of bond-related behaviors may therefore be differently involved in the transition of attachment from the father to the partner.

**Fig. 2:**
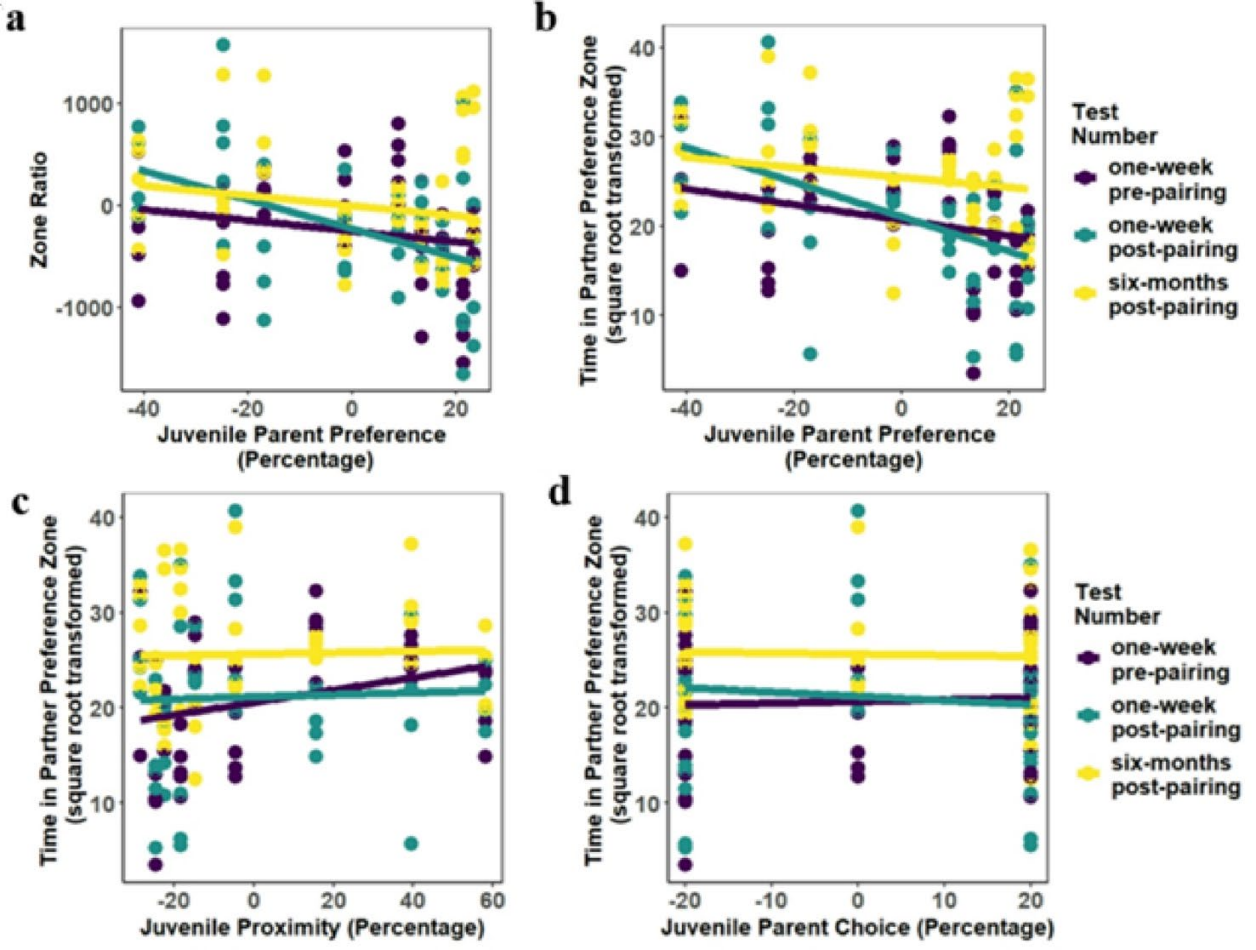
The main effects of (**a**) *Juvenile Parent Preference* on Zone Ratio, and the main effects of (**b**) *Juvenile Parent Preference*, (**c**) *Juvenile Proximity*, and (**d**) *Juvenile Choice* on Partner Zone duration. (**a**) Zone Ratio results suggest females that spent more time in proximity to their parents during juvenile preference testing (*Juvenile Parent Preference*) exhibited a greater preference for the father over the partner during the present adult testing (more negative Zone Ratio scores indicate a greater preference for the father over the partner). (**b**) Females that spent more time in proximity to their parents during juvenile preference testing (*Juvenile Parent Preference*) spent less time in proximity to their partners during the present adult testing. (**c**) Females that spent more time in proximity to their fathers during the reunion period following social separation testing as juveniles (*Juvenile Proximity*) spent more time in proximity to their partner during these adult preference tests. (**d**) Females that choose their parents more during catch and release as juveniles (*Juvenile Choice*) spent more time in proximity to their partners during this adult test. *Juvenile Parent Preference* (**a** & **b**) represents time in proximity over a three-hour testing period, whereas *Juvenile Proximity* (**c**) and *Juvenile Choice* (**d**) represent female’s proximity behavior following a separation from the parents (during previous juvenile social separation testing and juvenile catch and release testing). Points and lines on the graph are colored by test number (one-week pre-pairing, one-week post-pairing, and six-months post-pairing) for visualization purposes; however, the model results indicate an overall effect of these three bond-related behaviors on time in proximity to the partner across the three tests.

Based on the results from the *Zone Ratio* model, females spent less time in proximity to their partner and more time in proximity to their father during this study’s preference tests if they exhibited greater time in proximity to their parents during juvenile preference testing (β = −7.78, SE = 2.28, *t =* −3.42, *p <*.001, *f*^*2*^ *=* 0.087, R^2^ = 0.1310; Fig. 2a, Supplementary Table S1a). Similarly, when specifically focusing on the *Partner Zone* model results, females that spent more time in proximity to their parents during juvenile preference testing spent less time in proximity to their partners during this adult testing (β = −0.155, SE = 0.030, *t =* −5.17, *p <*.001, *f*^*2*^ = 0.207; Fig. 2b). Interestingly, when examining the effects of a different measure of juvenile proximity preference within the same model, females that spent more time in proximity to their fathers during the reunion period following social separation testing as juveniles spent more time in proximity to their partner during these adult preference tests (β = 0.060, SE = 0.021, *t =* 2.85, *p =*.005, *f*^*2*^ = 0.062; Fig. 2c, Supplementary Table S1b). Based on a third measure of juvenile proximity preference, females that chose their parents more during catch and release as juveniles also spent more time in proximity to their partners during this adult test (β = 0.101, SE = 0.036, *t =* 2.81, *p =*.006, *f*^*2*^ = 0.060, R^2^ = 0.2580; Fig. 2d). Therefore, whereas the measure of juvenile proximity preference did not consistently explain preference for time near the partner, for juvenile measures of proximity following a brief separation (juvenile social separation and catch and release testing), greater juvenile father-daughter proximity preference predicted greater adult preference for the partner across all three preference tests. It should be noted that these effect sizes are relatively small. With regards to time spent near the father (*Father Zone*), there was a non-significant trend towards females spending more time in proximity to their fathers if they also spent more time in proximity to their parents during juvenile preference testing (β = 0.055, SE = 0.032, *t =* 1.71, *p =*.131, *f*^*2*^ = 0.068; Supplementary Fig. S7; Supplementary Table S1c).

With regards to time spent touching the partner’s grate, females that spent a greater amount of time in proximity to their parents during juvenile testing spent less time touching the partner’s grate during the one-week post-pairing test (β = −0.037, SE = 0.011, *t =* −3.40, *p <*.001, *f*^*2*^ = 0.161; Supplementary Fig. S8; Supplementary Table S1d). When examining time spent touching the father’s grate, females that exhibited a greater increase in time in proximity to their father during infant testing spent more time touching the father’s grate during the six-month post-pairing test (β = 0.030, SE = 0.009, *t =* 3.18, *p =*.002, *f*^*2*^ = 0.102; Supplementary Fig. S9a, Supplementary Table S1e). Similarly, females that exhibited greater separation distress during infant testing spent more time touching their father’s grate during the one-week post-pairing test (β = 0.008, SE = 0.004, *t =* 2.16, *p =*.033, *f*^*2*^ = 0.051; Supplementary Fig. S9b), but the effect size for this bond-related behavior did not meet our threshold of significance for interpretation based on our sensitivity analyses.

For time spent overall in either the father or partner zone (*Social Zone*) and time spent in the non-social areas of the testing arena (*Other Zone*), we did observe a few small effects (Supplementary Table S1f & S1g). However, the nuances of these results are hard to assign meaning to. Please see supplementary materials for all results.

### Experiment 2: prediction 1

For Experiment 2, we conducted a series of four [^18^F]FDG PET scans. Two scans were conducted while the female was an adult still living in her natal group (27–28 months in age) and two were conducted six months post-pairing (35–36 months in age). At each of the two time points, females were tested once with their current attachment figure (father in the natal group, partner post-pairing) and once following a 30-minute separation from their attachment figure. We limited our analyses to a small number of brain regions within an a-priori network of interest: the social salience network (amygdala, hypothalamus, lateral septum, nucleus accumbens, ventral pallidum, and ventral tegmental area), the periaqueductal gray, and cerebellum (Supplementary Fig. S2). We also examined whole brain glucose metabolism across the four conditions. Immediately before running each [^18^F]FDG PET scan, we collected a blood sample for cortisol analyses. We predicted that brain glucose metabolism (as measured by Total Activity from an [^18^F]FDG PET neuroimaging scan) would be higher at the six-month post-pairing time points compared to the one-month pre-pairing time points; however, we expected glucose metabolism to be lower during separation conditions compared to when females were tested with their current attachment figure (father during the pre-pairing tests and partner during the post-pairing tests). We also predicted plasma cortisol levels would be higher when females are separated from their current attachment figures. Interestingly, we did not find support for any of our predictions. Glucose metabolism was lower post-pairing in all brain regions assessed (social salience network, periaqueductal gray, cerebellum, and whole brain) and did not differ significantly between the stress buffered and separation distress conditions within a timepoint (one month pre-pairing and six-months post-pairing). We also did not find any differences in cortisol responses across all four test conditions.

**Fig. 3:**
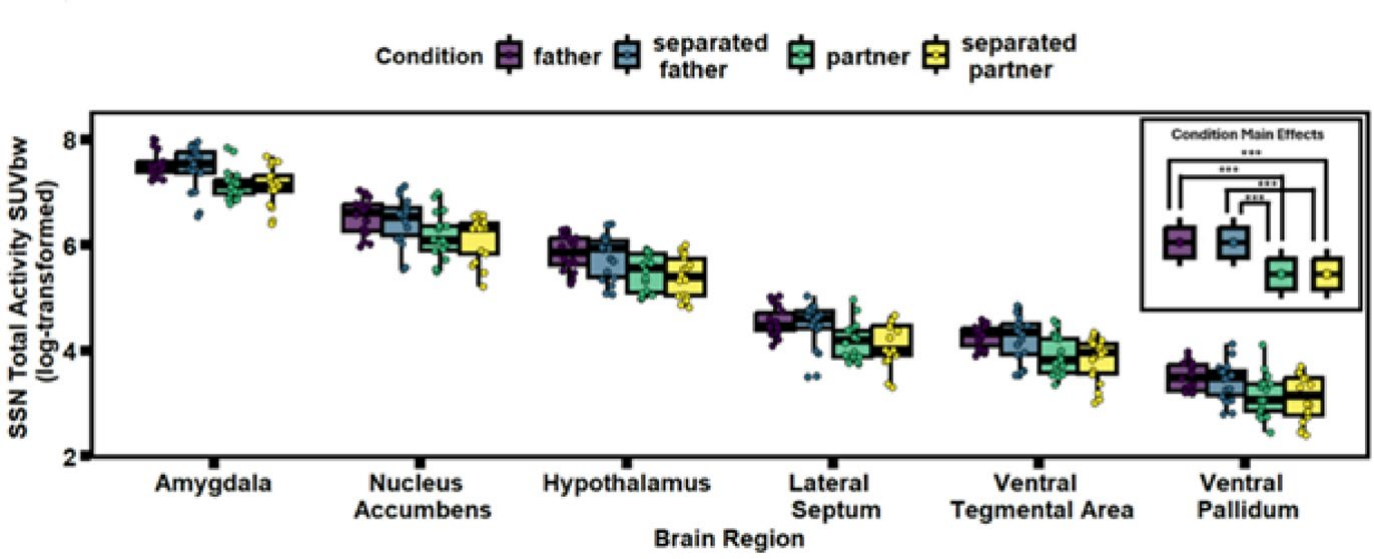
The main effect of *Condition* on Social Salience Network (SSN) glucose metabolism (SUVbw). Glucose metabolism in the SSN is higher during both pre-pairing tests (father and separated father) than during both post-pairing tests (partner and separated partner). Within the two time points (pre-pairing and post-pairing), glucose metabolism does not significantly differ between the stress buffered condition (father/partner) and the separation distress condition (separated father/partner). Data are shown for each of the six brain regions that make up the SSN. Analyses were completed using data from all six regions combined, and results are interpreted as effects of test condition on the SNN (not the separate brain regions). Legend in top right corner indicates the statistically significant pairwise comparisons between the four test conditions. father = when tested with father when still in natal group; separated father = when separated from father while still in natal group; partner = when tested with partner six-months post-pairing; separated partner = when separated from partner six-months post-pairing; SUVbw = Total Activity (glucose uptake) calculated as Standardized Uptake Value normalized by body weight; SSN = Social Salience Network. Significant differences for pairwise comparisons between tests indicated as: * < 0.05; ** < 0.01; *** < 0.001.

The best-fitting model for social salience network activity (R^2^ = 0.9712, *f*^2^ = 0.751; Supplementary Table S2a) suggested females exhibited less total activity when scanned with their partner (β = −0.365, SE = 0.034, *t =* −10.86, *p <*.001; Fig. 3) and separated from their partner (β = −0.421, SE = 0.034, *t =* −12.49, *p <*.001; Fig. 3), compared to when females are scanned with their fathers or separated from their fathers.

Similar to the results for the social salience network, periaqueductal gray activity was lower during both six-month post-pairing scans compared to when females were tested with their father while in their natal group one-month pre-pairing (R^2^ = 0.8253, *f*^2^ = 3.11; Supplementary Table S2b; Supplementary Fig. S10). We also found cerebellum activity was lower when females were tested with their partner and when they were separated from their partner compared to when they were tested with their father while in their natal group (R^2^ = 0.559, *f*^2^ = 1.27; Supplementary Table S2c; Supplementary Fig. S11). Compared to when they were tested with their father while in their natal group, whole brain activity was also lower when females were tested with their partner at the post-pairing time point (R^2^ = 0.7293, *f*^2^ = 2.61; Supplementary Fig. S12; Supplementary Table S2d). Across all brain regions tested for the present study, there was no significant difference in glucose metabolism between the stress buffered and separation distress conditions within the two time points, regardless of the current attachment figure (father when in natal group and partner when paired; Supplementary Table S2). We did not have enough evidence to suggest cortisol differed between any of the four test conditions (R^2^ = 0.730; Supplementary Table S2e; Supplementary Fig. S13).

### Experiment 2: prediction 2

We also assessed how measures of infant and juvenile bond-related behaviors impacted glucose metabolism across the four [^18^F]FDG PET scans. Five of the 12 measures from four historical experiments and scan samples significantly explained variability in adult glucose metabolism: (1) percentage of time juveniles spent in proximity, contact, or tail-twining with their fathers following a 30-minute separation test (*Juvenile Proximity*), (2) percentage of time our subjects’ parents spent in affiliative contact during scan samples collected over the first 14 months of our subjects’ lives (*Parent Affiliation*), (3) percentage of time infants spent touching the grate separating them from their father during an infant open field test (*IOF Grate*), (4) percent change in vocalizations when separated from the father compared to when tested with the father during a juvenile social separation test (*Juvenile Vocalizations*), and (5) percentage of times juveniles chose their parents over strangers following a brief separation during a catch and release test (*Juvenile Parent Choice*).

We predicted higher expression of bond-related behaviors would result in enhanced reduction of glucose metabolism during separation conditions if attachment figures were buffering females from stress. Our results generally supported this prediction, but it varied based on the measure of bond-related behavior. For example, our measure of juvenile female proximity to the father following a brief separation (*Juvenile Proximity*) was a significant predictor of variability across all brain regions tested, with higher juvenile proximity predicting lower glucose metabolism. However, if females observed their parents displaying a greater level of affiliation during the first 14 months of their lives (*Parent Affiliation*), glucose metabolism was higher in the social salience network and periaqueductal gray. Therefore, different measures of bond-related behaviors differently explained variability in glucose metabolism across all four tests. We did not have enough evidence to suggest interactions between test condition and bond-related variables significantly explained variability in plasma cortisol.

With regards to the effect of expression of father-daughter bond-related behaviors on glucose metabolism within the social salience network (R^2^ = 0.971; Supplementary Table S2a), we found significant interaction effects between *Condition* (pre-pairing with father, pre-pairing separated from father, post-pairing with partner, post-pairing separated from partner) and expression of bond-related behaviors. Specifically, *Juvenile Proximity* was negatively related to Total Activity when females were tested with their partner (β = −0.006, SE = 0.001, *t =* −4.85, *p <*.001) and separated from their father (β= −0.012, SE = 0.001, *t =* −10.22, *p <*.001), suggesting females that spent a greater percentage of time in proximity to their fathers following a separation as juveniles exhibit lower activity of the social salience network during these two adult test conditions (*f*^*2*^ *=* 0.228; Supplementary Fig. S14a). We found a positive relationship between *Parent Affiliation* and Total Activity when females were with their partners (β = 0.022, SE = 0.005, *t =* 4.79, *p <*.001) and separated from their partners (β = 0.017, SE = 0.005, *t =* 3.72, *p <*.001), suggesting they exhibited higher activity of the social salience network during these two conditions if their parents spent a greater percentage of time in affiliative contact when they were juveniles (*f*^*2*^ *=* 0.096; Supplementary Fig. S14b).

**Fig. 4:**
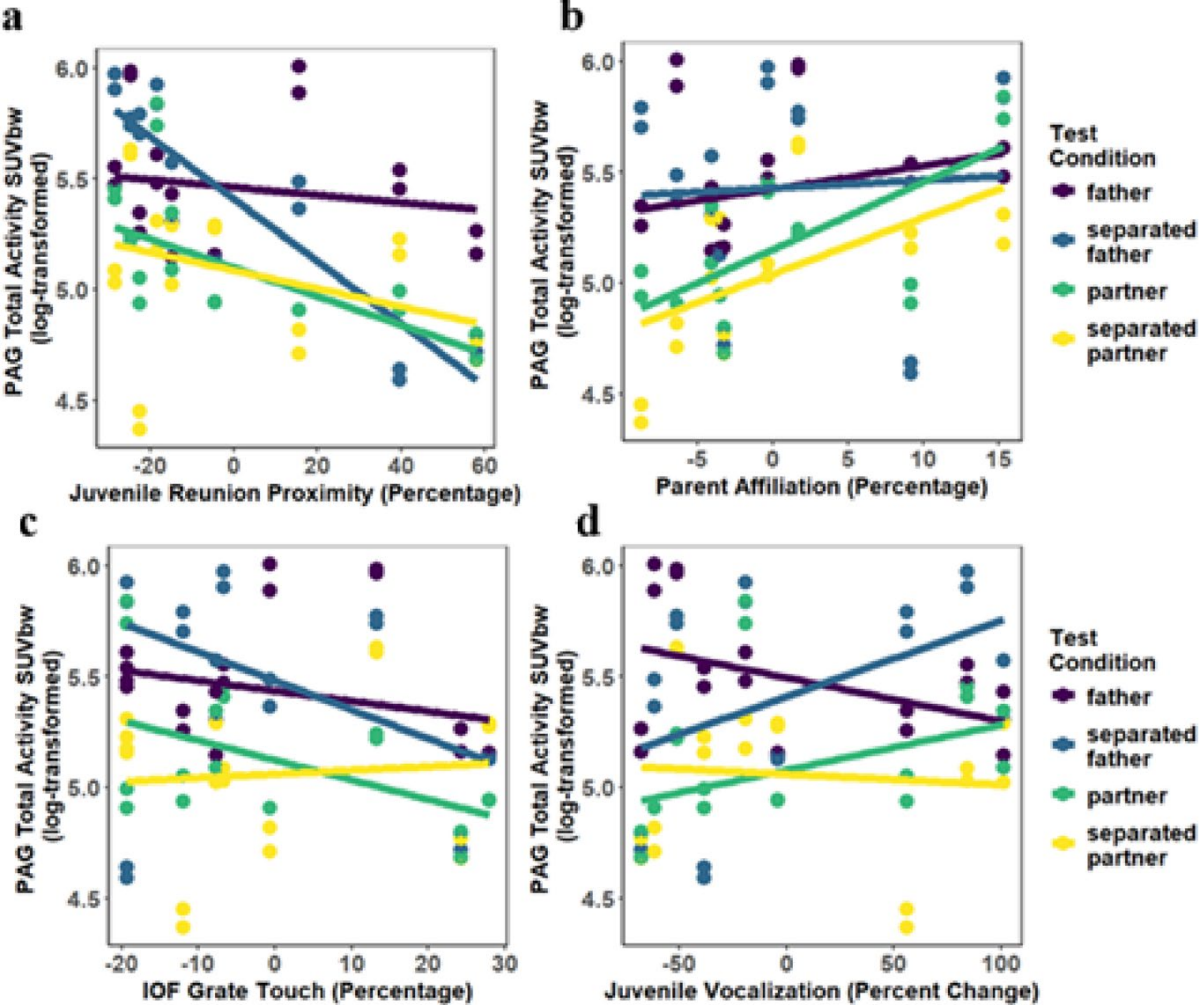
The interaction effect between *Condition* and (**a**) *Juvenile Proximity*, (**b**) *Parent Affiliation*, (**c**) *IOF Grate Touching* and (**d**) *Juvenile Vocalization* on periaqueductal gray glucose metabolism (SUVbw). (**a**) Females that spent a greater amount of time in proximity to their fathers following juvenile separation testing (*Juvenile Proximity*) exhibit lower periaqueductal gray activity when separated from their father in the present testing. (**b**) Females exhibit greater periaqueductal gray activity in both post-pairing conditions if their parents spent a greater percentage of time in affiliative contact when subjects were infants and juveniles (*Parent Affiliation*). (**c**) If females spent a greater percentage of time touching their father’s grate during Infant Open Field (IOF) testing as infants (*IOF Grate*), they had lower periaqueductal gray activity when tested with their partner but higher periaqueductal gray activity when separated from their partner. (**d**) Females that vocalized more when separated from their fathers as juveniles (*Juvenile Vocalization*) exhibit greater periaqueductal gray activity when with their partners and separated from their fathers, but lower periaqueductal gray activity when separated from their partner. father = when tested with father when still in natal group; separated father = when separated from father while still in natal group; partner = when tested with partner six-months post-pairing; separated partner = when separated from partner six-months post-pairing; SUVbw = Total Activity (glucose uptake) calculated as Standardized Uptake Value normalized by body weight; PAG = Periaqueductal gray.

When examining our results for the periaqueductal gray (R^2^ = 0.825; Supplementary Table S2b), we found several significant interaction effects between *Condition* and bond behavior-expression variables. Specifically, we found a negative relationship between *Juvenile Proximity* and Total Activity when females were tested separated from their fathers (β = −0.009, SE = 0.003, *t =* −3.21, *p =*.002), suggesting females that spent a greater amount of time in proximity to their fathers following juvenile separation testing exhibited lower periaqueductal gray activity when separated from their father (*f*^2^ = 0.234; Fig. 4a). We also found a significant interaction between *Condition* and *Parent Affiliation* (*f*^2^ = 0.422). Females exhibited greater periaqueductal gray activity when tested with their partners (β = 0.044, SE = 0.011, *t =* 4.08, *p <*.001; Fig. 4b) and separated from their partners (β = 0.054, SE = 0.011, *t =* 5.06, *p <*.001; Fig. 4b) if their parents spent a greater percentage of time in affiliative contact when subjects were infants and juveniles. Interestingly, we found opposite relationships between Infant Open Field (*IOF*) *Grate* and Total Activity depending on whether females were tested with their partner or separated from their partner (*f*^2^ = 0.275; Fig. 4c). If females spent a greater percentage of time touching their father’s grate during IOF testing as infants, they had lower periaqueductal gray activity when tested with their partner (β = 0.010, SE = 0.005, *t =* 2.09, *p =*.042) but higher periaqueductal gray activity when separated from their partner (β = 0.023, SE = 0.005, *t =* 4.76, *p <*.001). Interactions between *Condition* and *Juvenile Vocalizations* also significantly explained variability in periaqueductal gray activity (*f*^2^ = 0.240; Fig. 4d). Specifically, we found a positive relationship between *Juvenile Vocalization* and Total Activity when females were tested with their partner (β = 0.006, SE = 0.002, *t =* 4.24, *p <*.001) and separated from their father (β = 0.003, SE = 0.002, *t =* 2.20, *p =*.032), but a negative relationship when females were separated from their partner (β = 0.006, SE = 0.002, *t =* 3.94, *p <*.001). These findings suggest females that vocalize more when separated from their fathers as juveniles exhibit greater periaqueductal gray activity when with their partners and separated from their fathers, but lower periaqueductal gray activity when separated from their partner. Taken together, these four different measures of bond-related behaviors uniquely contribute to variability in females’ neural responses to our test conditions, with bonding behaviors correlating with a decrease (e.g., *Juvenile Proximity*), increase (e.g., *Parent Affiliation*), or condition-dependent change in glucose metabolism (e.g., *IOF Grate* correlating with lower metabolism with the partner and higher metabolism when separated from the partner).

We found a negative relationship (*f*^*2*^ = 0.239; Supplementary Fig. S15) between *Juvenile Proximity* and Total Activity in the cerebellum when females were tested separated from their fathers (β = −0.012, SE = 0.004, *t =* −2.77, *p =*.010, R^2^ = 0.559; Supplementary Table S2c), suggesting females that spent a greater amount of time in proximity to their fathers following juvenile separation testing exhibited lower cerebellum activity when separated from their father.

When examining variability in whole brain activity, we found significant interaction effects between *Condition* and two of the bond behavior-expression variables in our best-fitting model (R^2^ = 0.729; Supplementary Table S2d). Specifically, we found a negative relationship between *Juvenile Proximity* and Total Activity when females were tested with their partners (β = −0.007, SE = 0.003, *t =* −2.35, *p =*.023) and separated from their fathers (β = −0.014, SE = 0.003, *t =* −4.48, *p <*.001), suggesting females that spend a greater amount of time in proximity to their fathers following juvenile separation testing exhibited lower whole brain activity in these two conditions (*f*^2^ = 0.342; Supplementary Fig. S16a). We found a negative relationship between *Juvenile Parent Choice* and Total Activity when females were separated from their partners (β = −0.010, SE = 0.004, *t =* −2.27, *p =*.028), suggesting females that choose their parents a greater percentage of time during catch and release testing have lower whole brain activity when separated from their partners (*f*^2^ = 0.109; Supplementary Fig. S16b). Both juvenile tests (*Juvenile Proximity* and *Juvenile Parent Choice*) represent a preference for proximity with the father following a temporary separation.

Plasma cortisol did not significantly differ across our four test conditions (R^2^ = 0.730; Supplementary Table S2e; Supplementary Fig. S13), but variability may be explained by interactions between *Condition* and *Infant Proximity.* Females had significantly higher levels of cortisol when separated from their partners if they spent a greater percentage of their time being carried by their fathers for the first nine months of their lives (*Infant Proximity*); however, the effect size for this interaction did not exceed our threshold for reliable interpretation (β = −25.87, SE = 10.03, *t =* −2.58, *p =*.018; *f*^2^ = 0.212; Supplementary Figure S18).

## Discussion

To investigate how the expression of father-daughter bond-related behaviors were associated with both behavioral and neural correlates of social bonds, we conducted two parallel experiments on female titi monkeys. Experiment 1 examined how the expression of bond-related behaviors impacts proximity maintenance between the father and a new adult partner across three preference tests (one-week pre-pairing, one-week post-pairing, and six-months post-pairing). Experiment 2 investigated how expression of bond-related behaviors affects brain glucose metabolism during four [^18^F]FDG PET scans (one-month pre-pairing with father, one-month pre-pairing separated from father, six-month post-pairing with partner, six-month post-pairing separated from partner). In Experiment 1, we found that females shifted from preferring their father to preferring their partners after six months of pairing, and that expression of bond-related behaviors explained variability in these social preferences. In Experiment 2, we found overall glucose metabolism was generally lower post-pairing in all brain regions examined, and expression of infant and juvenile bond-related behaviors further explained variability in neural activity in response to pairing status and separation distress. Plasma cortisol did not significantly differ between tests, but variability may be explained by infant bonding behavior with the father. Below, we summarize these findings.

When Experiment 1 began, females were about 28 months old—near the age at which they start puberty (on average, 30 months^67)^ and would likely leave the natal group to form monogamous bonds in the wild^36,38,39,68^. By the end of the study (around 35 months old), each female had been paired with an adult male for six months: a period shown to yield stable partner preferences^64^. However, it remained unclear how differences in daughters’ attachments to their fathers, as measured by expression of father-daughter bond-related behavior, would impact their preference for maintaining proximity with their father once they formed a pair bond.

In the wild, titi monkeys do not typically emigrate far from their natal groups^69^, and offspring are not forced out by aggression^36,38^. Thus, some developmental or social mechanism likely motivates offspring to leave their fathers for an unfamiliar partner. Human studies similarly indicate that parent–child relationship quality can shape early adult romantic attachments^31–35^, so we hypothesized that a stronger father-daughter bond might influence how a female transitions to her first pair bond. Across the three preference tests—one-week pre-pairing, one-week post-pairing, and six-months post-pairing—females generally spent more time near their fathers during the preference paradigm early on but gradually shifted toward spending more time near their partners by six months post-pairing. Notably, however, the preference for the partner was weaker than the preference typically shown in comparisons between a partner and a stranger^13,64^. Even after six months without direct contact, females still appeared to maintain some attachment to their fathers. Importantly, those with higher paternal proximity scores in infant and juvenile tests spent comparatively less time with their partners, suggesting they were more likely to preserve their paternal bond. These findings challenge the notion that titi monkeys can maintain only a single selective attachment and raise questions about how long father-daughter attachments might persist alongside adult pair bonds.

Bond behavior-expression scores, especially those reflecting juvenile proximity behavior following a brief separation, predicted more time in the partner’s preference zone across all three time points; however, the effect sizes of these measures of bond expression were small. In contrast, high juvenile parent preference sometimes correlated with less partner proximity. While we recognize that expressions of proximity maintenance and separation distress are critical aspects of pair bonds^6^, it is possible that these two categories of behaviors expressed during early developmental periods may be differently associated with adult bonding behaviors. Our results suggest that greater separation distress as juveniles may prime females to spend more time in proximity to their partners as adults, whereas more time in proximity to the parents while still in the natal group may predict greater proximity to the father post-pairing. Regardless of the underlying factors driving individual differences in behavior, the overall patterns that females shifted to a greater preference for their partner while maintaining a similar amount of time near the father throughout the three tests^13,60^ suggests these females might be better at preserving multiple attachments simultaneously than previously assumed. Future research could address how father-daughter bonds evolve over longer periods, especially in naturalistic settings where ongoing interactions with the natal group remain possible.

One notable limitation of the present study is that females could not select their partners like they would in the wild. A recent study from our lab suggests that allowing titi monkeys to select their partners based on initial compatibility may further enhance affiliation between partners^70^. Interestingly, mate choice in wild titi monkeys appears to be more opportunistic rather than relatedness- or heterozygosity-based mate choice^71^, which would make our quasi-random selection of partners not too different from what occurs in the wild. It would be valuable to assess whether father-daughter bonds would differently impact relationships between pairs that choose each other and pairs that are assigned based on opportunistic availability. It is also important to explore how the partner’s relationship with his father as well as his general willingness to express bond-related behaviors may further impact adult relationships. Whereas lab studies offer the ability to assess longitudinal patterns of bond expression over the duration of a subject’s life, a notable limitation of this study is that, once females are paired, they cannot freely interact with their fathers as they would in the wild^69^. It would be important to examine whether these findings persist when paired females are allowed to still maintain interactions with their natal group in a natural environment.

Overall, Experiment 1 suggests that the expression of father-daughter bond behaviors lays a foundation for forming a strong pair bond, yet daughters may nevertheless continue to experience and nurture attachment to their father. This pattern underscores the possibility of overlapping mechanisms for filial and adult attachments in titi monkeys.

To explore the neural underpinnings of these bonds, we measured brain glucose metabolism (via [^18^F]FDG PET imaging) and plasma cortisol while females were still in their natal group (tested with father vs. alone) and again at six months post-pairing (tested with partner vs. alone). Prior studies on male titi monkeys showed increased whole-brain metabolism early in pair bonding^46,47^. Contrary to our prediction, we observed an overall decrease in glucose metabolism at six months post-pairing in females in all brain regions examined, possibly reflecting age-related changes or a different female-specific trajectory of metabolic activity^58^. While we are unable to disentangle the effects of pairing from the effects of aging in our present study, a previous study in another non-human primate showed only a 5% change in standard uptake value over six months^72^, and human studies have shown about a 0.2% per year decline in glucose metabolism measures from PET imaging^73,74^. Typical test-retest variability for PET imaging in general is around 5–7%, so these changes in metabolism with age were likely negligible over the small duration of our study (scans completed seven to nine months apart). Females were also all adults for the present study so they would not have been expected to experience major developmental periods within our testing period. In a previous titi monkey study, whole brain glucose metabolism did not significantly differ between 13 and 23 months of age (pre-pubescent ages), and slightly decreased from 23 to 33 months, but the subjects in that study were paired between the two scans, so it is not possible to distinguish between the effects of aging and pairing^58^.

Despite the general decrease in glucose metabolism, bond behavior-expression measures explained meaningful variation in neural activity. Females that spent more time near their fathers as juveniles exhibited lower social salience network—comprising the amygdala, hypothalamus, lateral septum, nucleus accumbens, ventral pallidum, and ventral tegmental area—and whole brain activity when separated from their father and when tested with the partner. This pattern is consistent with the idea that stronger social bonds can buffer stress, resulting in lower metabolic responses to separation^65,66^.

Conversely, observing high parental affiliation predicted higher social salience network activity in the post-pairing tests, suggesting that witnessing strong parental bonds might sensitize females to changes in their own social relationships. We also found parallels in the periaqueductal gray and cerebellum, where high father-daughter proximity predicted lower glucose metabolism during paternal separation. Notably, some measures of separation distress predicted the opposite effect in the periaqueductal gray, pointing to distinct functional roles for proximity maintenance versus distress behaviors. For instance, greater juvenile vocalization was linked to higher periaqueductal gray activity in certain contexts, aligning with the periaqueductal gray’s established role in separation distress and social pain^75^. In general, we observed greater variability in periaqueductal gray activity in the six-month post-pairing tests compared to the pre-pairing tests, suggesting this brain region may be more relevant for pair bonds than filial bonds. Previous research on titi monkeys^49^, humans^61^, and pair bonding rodents^62^ have similarly identified the role of the periaqueductal gray in adult bonds, whereas studies on the neural correlates of offspring attachment do not include activation of the periaqueductal gray^76–79^, making this brain region particularly interesting to focus on when disentangling pair bonding and filial bonding circuitry.

Taken together, these neural data suggest considerable overlap in the circuitry underlying filial and pair-bond attachments, lending further support to the idea that pair bonds may have evolved from parent-offspring bonds^50–53^. However, the exact neurochemical pathways—oxytocin, vasopressin, dopamine, or opioids—remain unclear. Future research measuring specific receptor binding or neurotransmitter release would clarify whether different aspects of bond behaviors modulate these systems in distinct ways.

We did not find evidence to suggest plasma cortisol significantly differed in response to our testing paradigm. However, females generally had higher levels of cortisol across all tests, and particularly when separated from the partner, if they spent a greater amount of time being carried by the father during the first nine months of their lives. In a previous study using a similar 30-minute separation paradigm, we similarly found no significant difference in cortisol levels between the stress buffered and separation distress conditions in juvenile female titi monkeys tested with and without their fathers^29^. In that same study we also found that measures of infant father-daughter bond-related behaviors explained variability in cortisol responses that mirror results from our present study. Witczak and colleagues found that females that spent more time in proximity to their fathers during infant open field testing at four months of age exhibited a greater rise in cortisol during juvenile (ages 14–18 months) separation testing^29^. Given the overlap in previous findings in juveniles and findings in adults in our present study, it is possible that early relationships between fathers and offspring have long-lasting impacts on titi monkey physiology. It should be noted that there are methodological constraints associated with cortisol measurements. Our measures may reflect the effects of capture and sedation, which may obscure the effects of our test conditions. The average time between capturing subjects and collecting blood samples was below the five-minute cutoff recommended for capturing the effects of test conditions^80^; however, we cannot rule out the possibility of these other experiences (e.g., capture, injection, sedation) impacting our plasma cortisol measures. It would be valuable to assess whether father-son bonds differently impact behavioral, neural, and physiological correlates of pair bonds in males.

It is important to acknowledge the limitations of our study. Our subjects were laboratory-housed titi monkeys, and we had a relatively small sample size (*N* = 9), so the results from this study may not replicate in the wild. Future studies should aim to replicate wild conditions, incorporate measures of father-son bond expression, better isolate the effects of aging and multiple stressors, and identify neurochemicals involved in proximity maintenance, stress buffering, and separation distress.

In conclusion, our two experiments reveal that expression of father-daughter bond-related behaviors are significantly associated with both the behavioral expression and neural correlates of female attachment in titi monkeys. Strong paternal bonds, particularly greater time in proximity to the father following short separations as a juvenile, predict greater time in proximity with the partner during preference testing, a robust foundation for pair bonding, and the potential to maintain aspects of the filial attachment even after pairing. Neuroimaging data indicate substantial overlap in the neural circuits supporting filial and adult attachments, though overall glucose metabolism may change with age, pair-bond duration, and/or expression of bond-related behaviors. These findings contribute to our understanding of the flexible, multi-layered nature of social bonding and underscore the importance of considering individual differences in the expression of bond-related behaviors.

## Methods

### Subjects and housing

Subjects were nine female titi monkeys (ages 27–36 months), their fathers (*N* = 9), and their vasectomized partners (*N* = 9). This age range represents a time when females are likely to emigrate from their natal group in the wild and form a pair bond^36,38,39,68^. Whereas the original population of titi monkeys was wild-born in the early 1970 s, all subjects used in the current study were born and housed at the California National Primate Research Center. Given the closed nature of this captive colony, we had to select partners for subjects based on genetic relatedness (using kinship pedigree analyses to ensure partners were < 25% related to each other^81)^ and eligibility to be paired at the time when the subject came of age for the present study. Males were either adults that were still living in their natal groups awaiting a partner or had been separated from their past partner for at least two weeks, which is the amount of time our lab has found is necessary for a titi monkey to be willing to form a new pair bond after losing a partner (K. Bales, unpublished communication).

Titi monkeys lived in their natal groups with their parents and any older siblings. Females lived with their partner once paired around 29 months. Families were housed in a 1.2 m x 1.2 m x 2.1 m–1.2 m x 1.2 m x 1.8 m stainless steel cage. They were fed twice daily with monkey chow, rice cereal, carrots, apples, and bananas. They were kept on a 12:12 light: dark cycle, with lights on at 0600 h and off at 1800 h. Room temperature was maintained at 21 °C. This housing setup matches that of previous studies^28,29^. This study was approved by the IACUC of the University of California, Davis (IACUC #19641 and #21445); and complied with legal requirements of the United States and the ARRIVE guidelines. No animals were sacrificed in the course of this research.

To study the expression of bond-related behaviors’ impacts on the behavioral correlates of proximity maintenance and the neural correlates of separation distress and stress buffering, we conducted two experiments simultaneously (Supplementary Fig. S1):

### Experiment 1 and 2 measures of bond-related behaviors

To examine the impact of father-daughter bond-related behaviors on behavioral and neurological responses during two experiments, we utilized methods developed in prior studies from our lab to quantify bond-related behaviors^29,30^. Three essential components of a pair bond are distress upon separation from the attachment figure^16^, preference for maintaining close social proximity to the attachment figure^13^, and affiliative partner-directed behaviors^6^. We used historical data collected by our lab to quantify infant and juvenile father-daughter bond-related behaviors as well as pair bond-related behaviors in adult pairs. We grouped these measures based on these three categories of behaviors important for bonds. For the present study, we analyzed data collected across several experiments and scan samples: an infant open field (IOF) test^41,82^, infant carry scan samples^83^, a juvenile social separation test^29^, a juvenile parent preference test^30^, and adult pair mate scan samples^84^.

### IOF test

When infants in our colony are four months old, they are placed in a novel arena for 20 min and separated from family members by a mesh grate^41,82^. Researchers rotate the subject’s family members (mother, father, and sibling) and an empty box at the grate every five minutes and film the infant’s reactions to the presence of the different stimuli. From the IOF test, we measured signs of separation distress (*IOF Locomotion* and *IOF Vocalization*) when females were separated from their fathers and proximity maintenance when with their fathers (*IOF Proximity* and *IOF Grate*) compared to when an empty box was placed at the grate. For more details, see Supplementary Materials (Supplementary Methods; Supplementary Table S4).

### Infant carry scan samples

From birth until nine months of age, we record where infants were in relation to their family members every two hours, five days per week^83^. Infants could be carried by their mother, father, sibling, or independently moving about the home-cage. In the present study we used these daily scan samples to measure the percentage of time females spent in proximity to the father in the home environment (*Infant Proximity).* For more details, see Supplementary Materials (Supplementary Methods; Supplementary Table S4).

### Juvenile social separation test

When all nine of our subjects were 14–18 months of age, they experienced a series of separation tests as part of a previous study^29^. For the present study, we focused on the saline control condition of the test. Briefly, females were given a 180 µl dose of saline intranasally, remained undisturbed with their family in the home-cage for 30 min, and then experienced one of two conditions: (1) both parents were removed and the daughter was left alone in the home environment for 30 min (separation distress condition), or (2) only the mother was removed and the daughter remained in the home-cage with her father for 30 min (stress buffering condition). Families were then reunited in the home environment. All nine subjects experienced both the separation condition and the stress buffering condition. We filmed behaviors both during testing conditions and in the 15 min following the end of testing. We used the juvenile social separation test to measure separation distress during testing (*Juvenile Vocalization* and *Juvenile Locomotion*) and time spent in proximity to the father following the end of the separation condition (*Juvenile Proximity*). For more details, see Supplementary Materials (Supplementary Methods; Supplementary Table S4).

### Juvenile parent preference test

All nine of our subjects experienced a series of parent preference tests from ages 18–20 months^30^. For the present study, we measured behaviors from the saline condition only. For this juvenile testing, females received an intranasal saline treatment (180 µl), remained undisturbed at home for 30 min, and then were moved to the center of our preference testing arena for approximately three hours. Female’s parents were on one side of the testing arena and a stranger pair of adult titi monkeys were on the other side of the arena. Females were separated from stimulus pairs by a grated window and could interact freely with either pair at the grates throughout the duration of the test. We recorded time spent in proximity to the parents, strangers, and within the non-social areas of the testing arena across five 30-minute observations. Following the three hour test, we performed five catch-and-release sessions, where we caught females from the center arena in a transport box, released them back into the center arena, and recorded which stimulus pair the females chose to stay in proximity to first (proximity needed to last at least 10 s to be indicated as a choice). From the juvenile parent preference test, we quantified preference for maintaining proximity to the parents during testing (*Juvenile Parent Preference*) and frequency of choosing the parents over a stranger following a brief separation period (*Juvenile Parent Choice*). For more details, see Supplementary Materials (Supplementary Methods; Supplementary Table S4).

### Adult pair-mate scan samples

Our lab collects adult pair-mate scan samples on every pair in our colony for the duration of their entire pairing^84^. Every two hours, five days per week, we record where pair-mates are in relation to each other. If partners are more than one arm-length apart, we record that as no proximity, but if they are physically close, we record the type of affiliation observed (proximity, contact, or tail-twining). For the present study, we used these data to calculate the percentage of observations pairs were observed displaying some form of affiliation (proximity, contact, or tail-twining) out of all observations recorded. We measured affiliation between parents observed by the daughter while she was in her natal group for the first 14 months of her life (*Parent Affiliation*) and affiliation between the female and her partner during their six months of pairing (*Pair Affiliation*) to quantify affiliative partner-directed behaviors. For more details, see Supplementary Materials (Supplementary Methods; Supplementary Table S4).

### Present study bond-related behaviors in data analyses

For data analysis, all measures of bond-related behaviors were centered about the mean value for our nine subjects. This allowed us to determine how variation in expression of bond-related behaviors is associated with various outcomes in our two experiments. For example, we could examine how females that spent a greater proportion of time in proximity to their fathers as infants differ from females that spent comparatively less time maintaining proximity with their fathers. To test our hypotheses, we defined higher expression of infant and juvenile father-daughter bond-related behaviors as greater relative expression of separation distress (*IOF Locomotion*,* IOF Vocalization*,* Juvenile Locomotion*,* Juvenile Vocalization*), proximity maintenance (*Juvenile Proximity*,* Juvenile Parent Preference*,* Juvenile Parent Choice*,* IOF Proximity*,* IOF Grate*,* Infant Proximity*) and affiliation (*Pair Affiliation*,* Parent Affiliation*). For more details on how each measure was quantified, see previous studies by Witczak and colleagues^29,30^ and the Supplementary Methods of the present study (Supplementary Table S4).

### Experiment 1 data collection

To assess behavioral preference for one attachment figure over another, females were tested in a total of three preference tests^13^. For all three tests, subjects were released into the center chamber of the three-chambered testing apparatus (Supplementary Fig. 18). Tests lasted for approximately 3 h, and we live-scored five consecutive 30-minute blocks (five *Observations* per preference test). The first preference test occurred when the female was still in her natal group one-week pre-pairing. Her father was on one side, with an unfamiliar male (with whom she was later paired) on the other side. Testing was repeated one-week post-pairing and six-months post-pairing (Supplementary Fig. S1). The side that stimulus animals were on alternated between tests to avoid development of a side preference.

All tests were video-recorded and live-scored using Behavior Tracker (www.behaviortracker.com) using an established ethogram (Supplementary Table S3). We quantified the amount of time a subject spent in the preference zone of their partner and their father, and the amount of time females spent touching the stimulus animals’ grates. We created a *Zone Ratio* score by multiplying the time females spent in their partner’s preference zone by + 1, the time in the father’s preference zone by −1, and the time in the neutral zone by 0, and summing these three values per observation. Positive values represented more time in the partner’s preference zone whereas negative values represented more time in the father’s preference zone. Values closer to zero either indicated a lack of choice between the father and partner, with the female spending relatively the same amount of time in each zone, or a preference for the non-social areas of the testing arena. To disentangle this lack of choice from spending equal amounts of time in both preference zones, we also measured overall time in either social zone (father’s or partner’s) and overall time in the non-social parts of the testing arena.

### Experiment 1 data analysis

All analyses were conducted in R Statistical Software (version 4.0.3, R Core Development Team, 2020). We performed a Shapiro Wilk test of normality and transformed non-normally distributed variables^85^. All tests were two-tailed and the significance threshold of 0.05.

We first identified which of the 12 bond behavior expression variables (*IOF Locomotion*,* IOF Vocalization*,* Juvenile Vocalization*,* Juvenile Locomotion*,* Juvenile Proximity*,* Juvenile Parent Preference*,* Juvenile Parent Choice*,* Infant Proximity*,* IOF Proximity*,* IOF Grate*,* Pair Affiliation*,* Parent Affiliation;* for more details see previous studies by Witczak and colleagues^29,30^, and Supplementary Methods of the present study; Supplementary Table S4) best explained variance in our outcome variables (Supplementary Table S3). To identify best-fitting bond behavior expression variables, we ran stepwise regression using the *leaps* package^86^. This method allowed us to iteratively add and remove variables in the predictive model to identify which subset of variables resulted in the model with the lowest prediction error^87,88^. To simplify the stepwise regression models, we first ran separate stepwise regression models for separation distress (*IOF Locomotion*,* IOF Vocalization*,* Juvenile Locomotion*,* Juvenile Vocalization*), proximity maintenance (*Juvenile Proximity*,* Juvenile Parent Preference*,* Juvenile Parent Choice*,* IOF Proximity*,* IOF Grate*,* Infant Proximity*) and affiliation (*Pair Affiliation*,* Parent Affiliation*) variables. Once we identified the top separation distress, proximity maintenance, and affiliation variables, we ran a final stepwise regression model that just included those top variables from each category (separation distress, proximity maintenance, affiliation), selecting the most theoretically relevant variables if any were highly correlated (Supplementary Table S5). The combination of bond/behavior expression variables that was identified as producing a model with the lowest prediction error was then used in our mixed-effects models (see Supplementary Methods for further model details).

We ran general linear mixed-effects models (LMM) using the *lmerTest* package^89^, with animal identity as a random effect to account for repeated measures. In our full model, fixed effects included *Test Number* (one-week pre-pairing, one-week post-pairing, six-months post-pairing), *Observation Number* (the five 30-minute time-blocks scored within each 3-hour preference test), *Partner Experience* (whether the male partner had previously been paired with another titi monkey [experienced] vs. not [naïve]), bond behavior-expression variables (identified by previous stepwise regression analyses), and interaction effects between *Test Number* and each bond behavior-expression variable. To determine the best-fitting model, we used backwards selection to remove any non-significant fixed effects^90^. We used a log likelihood ratio test to compare model fit to determine whether removing any non-significant fixed effects resulted in a better fitting model^91^ (Supplementary Table S6). The one final model represented the most likely hypothesized relationship between parameters given the data. When *Test Number* was statistically significant in our final model, we used the *eemeans* package^92^ to conduct pairwise comparisons between the three preference tests with Tukey’s post-hoc corrections. When final models included interaction effects, we assessed contrasts between conditional marginal means in the presence of interactions^88^. For all significant predictors we also calculated Cohen’s *f*^*2*^ as a measure of effect size^93,94^. Based on Cohen’s^95^ guidelines, *f*^*2*^ *≥* *0.02*,* f*^*2*^ *≥* *0.15*, and *f*^*2*^ *≥* *0.35* represent small, medium, and large effect sizes, respectively. We performed a sensitivity analysis using G*Power 3 prior to the main analysis to determine the minimum effect size (Cohen’s *f*^*2*^) that we could reliably interpret for each model. We interpreted results only for predictors that had an alpha of *≤* 0.05 and an effect size larger than that which we could interpret based on our sensitivity analysis.

### Experiment 2 data collection

In tandem with Experiment 1, females were tested in a total of four [^18^F]PET scans: two one-month pre-pairing while she was in her natal group and her primary attachment figure was her father, and two six-months post pairing, when females demonstrate a clear preference for their partner over strangers^64^. During the pre-pairing scans, we examined glucose metabolism when the female was scanned with her father (baseline), and after she was separated from her father for 30 min, to measure the neural correlates of distress upon separation from her primary attachment figure (for similar methods, see Hinde and colleagues^49^. Similarly, during the post-pairing scans the female was scanned with her partner and after a 30-minute separation from her partner. All [^18^F]PET scans were counter-balanced so five females were scanned with their current attachment figure first and four females were scanned separated from their current attachment figure first (Supplementary Fig. S1). After completing all four scans, we conducted one structural magnetic resonance imaging (MRI) scan to use for co-registration and quantification of [^18^F]FDG uptake ([^18^F]FDG; PETNET Solutions, Sacramento, CA, USA). [^18^F]FDG uptake has previously been used in titi monkeys as an approximation of brain activity^45,46,49,58^.

Females and their families were relocated to the testing room 48-hours prior to the start of the scan to reduce the effects of being in a novel environment on neural activity^49^. Titi monkeys were fasted for 10 h prior to the start of each PET scan, with water available *ad libitum*. On the day of the PET scan, females received a bolus [^18^F]FDG injection into the saphenous vein. The father remained in the testing cage during the “baseline” condition or was removed from the room while the female received her [^18^F]FDG injection during the “separation” condition. The mother and any siblings were removed from the testing room in both conditions when the female received her [^18^F]FDG injection. The female was returned to the testing cage (where she was either alone or with her father) and filmed for 30 min. Following the 30-minute uptake period, the females were hand-caught and sedated with ketamine (25 mg/kg IM). As soon as females were sedated, a 1.0 ml blood sample was collected via femoral venipuncture. We aimed to collect blood samples within five minutes of capture so plasma cortisol would reflect the effects of the separation or stress buffering condition, rather than the effects of capture and sedation^80^. Mean time from capture to blood sample collection was 4 min and 44.36 s (SD = 2:23.53; range = 2:16.00–12:06.00). Eight of the 36 blood samples were collected after the five-minute cutoff, but they were not outliers in our dataset, so we kept them in our analyses. Following blood collection, samples were placed on ice immediately, centrifuged at 1,610 x *g* at 4 °C, and the plasma extracted and stored at −80 °C until assay.

PET imaging was performed on the πPET dedicated brain scanner (Brain Biosciences, Rockville, MD). Anesthesia was maintained throughout the 60-minute scan with isoflurane. MRI scans were conducted in a GE Signa LX 9.1 scanner (General Electric Corporation, Milwaukee, WI, USA) with a 1.5 T field strength and a 3” surface coil. Region of interest (ROI) structures were drawn on each subject’s MRI image using PMOD (version 4.2) software (https://www.pmod.com/web/) using the “view” tool (Supplementary Fig. S19). ROIs for the present study were regions within the social salience network (amygdala, hypothalamus, lateral septum, nucleus accumbens, ventral pallidum, and ventral tegmental area), the periaqueductal gray, the cerebellum, and whole brain. The brain regions for the social salience network were identified based on their role in prairie vole pair bonding^57^, and their relevance to juvenile and adult titi monkey attachment relationships^45,58^. The periaqueductal gray and cerebellum were included due to their identified role in male titi monkey separation distress^49^. For hypothesized connections between the social salience network, periaqueductal gray, and cerebellum, see Supplementary Fig. S2. PET scan data were then co-registered with the same MRI image for each subject using the “fusion” tool in PMOD. To analyze ROI activity, we extracted the total activity for each ROI (left and right), which was calculated as Standardized Uptake Value normalized by body weight (SUVbw).

An enzyme immunoassay validated for titi monkeys^96^ was used to estimate plasma cortisol concentrations from blood samples. A total of two plates were assayed, with intra-assay CVs of 12.2% and 10.7%, with an inter-assay CV of 1.7%.

Females were then paired and remained with this partner for the duration of testing. Following six months of pairing, females were tested using the same paradigm as described above; however, they were tested with their partner or alone (separation condition).

### Experiment 2 data analysis

Data analyses for Experiment 2 were nearly identical to those conducted in Experiment 1. We checked for normality and transformed any non-normally distributed variables. We then conducted stepwise regression to identify which bond behavior expression variables we should include in our LMM analyses. All LMM analyses included ID as a random, repeated measure. In our full model for Social Salience Network, fixed effects included *Condition* (father, separated from father, partner, separated from partner), *Region* (amygdala, hypothalamus, lateral septum, nucleus accumbens, ventral pallidum, ventral tegmental area), *Side* (left, right), bond behavior expression variables (identified by previous stepwise regression analyses), and interaction effects between *Condition* and each bond behavior expression variable. We assessed patterns within the social salience network, rather than assessing each individual region within the network, because previous work has found that behavior may be most strongly linked to patterns of activity across a network, rather than within individual regions^56^. For our two regions outside of the social salience network (the periaqueductal gray and the cerebellum), we ran separate models for the specific regions. LMM analyses for whole brain and periaqueductal gray were the same but did not include *Region* as a fixed effect. LMM analyses for cerebellum and cortisol were the same as whole brain and periaqueductal gray but did not include *Side* as a fixed effect. We used backwards selection and a log-likelihood ratio test to identify the most parsimonious model that best explained variability in our data (Supplementary Table S7) and interpreted significance only from that one final model when *p <*.05 and effect size (Cohen’s *f*^2^) was above the threshold we could confidently interpret based on sensitivity analyses. When *Condition* was statistically significant in our final model, we used the *eemeans* package^92^ to conduct pairwise comparisons between the four [^18^F]PET scan conditions with Tukey’s post-hoc corrections. When final models included interaction effects, we assessed contrasts between conditional marginal means in the presence of interactions^81^. For more details regarding model decisions, see Supplementary Methods.

## Supplementary Information

Below is the link to the electronic supplementary material.

Supplementary Material 1

## Data Availability

The datasets generated during and/or analyzed during the current study are available in the Zenodo repository, https://doi.org/10.5281/zenodo.15660221.
