# Supplementary material for: The expression of father-daughter bond behaviors influences adult partner attachment in titi monkeys
Source: Sci Rep. 2025 Dec 16;16:1653. doi: 10.1038/s41598-025-31143-6 (PMC12799604; doi:10.1038/s41598-025-31143-6)
Supplement: Supplementary file 1 — Supplementary Material 1 [file 41598_2025_31143_MOESM1_ESM.pdf]

The expression of father-daughter bond behaviors influences adult partner attachment in titi monkeys

Lynea R. Witczak*[1,2], Allison R. Lau[1,2,3], Brad A. Hobson[2,4], Sara M. Freeman[5], Pauline B. Zablocki-Thomas[2], Madison Dufek[2], Emilio Ferrer[1], Abhijit J. Chaudhari[2,4,6], Karen L. Bales[1,2,3,7]

[1]Department of Psychology, University of California, Davis, Davis, CA

[2]California National Primate Research Center, University of California, Davis, Davis, CA

[3]Graduate Program in Animal Behavior, University of California, Davis, Davis, CA

[4]Center for Molecular and Genomic Imaging, University of California, Davis, Davis, CA

[5]Department of Biology, Utah State University, Logan, UT

[6]Department of Radiology, University of California, Davis, Davis, CA

[7]Department of Neurobiology, Physiology, and Behavior, University of California, Davis, Davis, CA

Corresponding Author: Lynea R. Witczak, Department of Biology, 100 Campus Drive, Elon, NC 95616. Ph: (240) 505-1309. Email: lwitczakoldfather@elon.edu

SUPPLEMENTARY METHODS

Quantification of Father-Daughter Bond-Related Behaviors

We have access to several measures of infant and juvenile father-daughter bond-related behaviors in our captive titi monkey colony. For the present study, we quantified several indicators of separation distress and proximity maintenance for our 9 subjects (Supplementary Table S4). Measures were collected both as scan samples and as responses to social challenges when females were infants and juveniles. For data analysis, all measures of bond-related behaviors were centered about the mean value for our 9 subjects. This allowed us to determine how variation in expression of bond-related behaviors influences various outcomes in our testing paradigm. For example, we could examine how females that spend a greater proportion of time in proximity to their fathers as infants differ from females that spend comparatively less time maintaining proximity with their fathers. Below are details regarding data collection for each measure of father-daughter bond-related behaviors:

IOF Locomotion

All infant titi monkeys at the California National Primate Research Center undergo infant open field (IOF) tests when they are four months of age. This test measures responses to a novel environment and reactions to the presence of family members during a stressful experience. Subjects are placed in the center of a one meter by one meter arena with a six-by-six square floor grid. One wall included a wire mesh grate that allowed auditory, visual, and olfactory access to a stimulus animal that was placed in a transport box on the other side of the grate. During this testing, subjects experienced four five-minute trials, each with a different stimulus on the other side of the grate (their father, their mother, their oldest sibling [if they had any], and an empty

box). For a more detailed description of the infant open field methods, see Larke and colleagues (Larke et al., 2017) and Savidge and Bales (Savidge & Bales, 2020). During testing, we count the number of lines that the infant crosses within each five-minute trial (defined as two or more limbs crossing a line; Larke et al., 2017). For the present study, we calculated the number of line crosses for each subject during the empty box condition and the father condition. We then calculated the percent change in line crosses from the father condition to the empty box condition ([{line crossing when alone – line crossing with father} / line crossing with father] * 100). We interpreted a greater increase in line crossing to indicate heightened distress upon separation, which may be an indicator of a higher expression of bond-related behaviors.

IOF Vocalization

During IOF testing we also score infant vocalizations. During this test we typically only hear infants make trill calls (as defined by Clink et al., 2019), but if they made any other separation distress calls, we combined those with the number of trills scored to create a total vocalization score. We then calculated the percent change in vocalizations from the father condition to the empty box condition. A greater increase in vocalizations was interpreted as an enhanced separation distress response, which is assumed to be an additional indicator of a higher expression of father-daughter bond-related behaviors.

Juvenile Locomotion

We quantified measures of juvenile bond-related behaviors using behaviors scored during the saline condition of our social separation testing described by Witczak and colleagues (Witczak et al., 2023). For this measure, we only assessed when juvenile females (ages 14-18 months) were treated with saline. Briefly, females were given a saline intranasal treatment,

returned to their family for 30 minutes, and then experienced one of two tests: 1) mother is removed from the home-cage for 30 minutes while the subject is left at home with her father, or 2) both parents are removed from the home cage-cage and the subject is left alone in the home cage for 30 minutes. We quantified the duration of time females spent locomoting when females were tested alone (separation distress condition) and when they were tested with their father (stress buffering conditions) and calculated the percent change in locomotion from the stress buffering to the separation distress condition. We interpreted a greater increase in locomotion as a heightened separation distress response, which may be indicative of a higher expression of father-daughter bond-related behaviors.

Juvenile Vocalization

We also quantified percent change in separation distress vocalization frequency from the stress buffering to the separation distress conditions of the social separation test described by Witczak and colleagues (Witczak et al., 2023) when females were treated with saline. The values for this measure were much larger than the values for all other bond-related variables; therefore, we rescaled this measure by dividing by 10 to make this variable on a more similar scale to our other predictors. A greater increase in vocalizations from the stress buffered to the separation distress condition represented an elevated stress response, which we interpreted as a higher expression of bond-related behaviors.

Juvenile Proximity

We also quantified measures of juvenile proximity maintenance using video recordings from our social separation testing (described in Witczak et al., 2023). Females were reunited with their families in their home-cage immediately following blood sample collection, and their

interactions were recorded for 15 minutes. For this measure, we only looked at behaviors during the reunion period following the saline condition of the separation distress condition (when females were left alone in the home-cage for 30 minutes). We calculated the duration of time that females spent in proximity, contact, or tail-twining with their fathers during this 15-minute reunion period (for detailed definitions of these behaviors, see Karaskiewicz et al., 2021)). We calculated the percentage of time females were in social proximity with their fathers as the total time in social proximity/contact/tail-twining out of the entire 15-minute reunion period ([duration of time in contact/proximity/tail-twining / 600 seconds] * 100). We interpreted a greater percentage of time maintaining social proximity to the father as a higher expression of father-daughter bond-related behaviors.

Juvenile Parent Preference

We quantified additional measures of juvenile proximity maintenance using data from the saline condition of parent preference testing described Witczak and colleagues (Witczak et al., 2024). We calculated the percentage of time females spent in the preference zone of their parents during the entire 3-hour parent preference test by dividing the total time in the parent zone by 150 minutes. We interpreted a greater percentage of time in the parents' preference zone as an indication of a higher expression of bond-related behaviors.

Juvenile Parent Choice

We also used data from the catch and release sessions (described in Witczak et al., 2024) when females were treated with saline. We calculated the percentage of times females chose their parents during the five catch and release bouts by dividing the number of times the female chose

her parents by five. We assumed females that chose their parents more often had a higher expression of father-daughter bond-related behaviors.

IOF Proximity

In addition to quantifying measures of separation distress during IOF testing, we were also able to quantify measures of infant proximity maintenance. We used the same definition of grate proximity that Larke and colleagues (Larke et al., 2017) used to quantify percent time infants spent in close proximity to the grate in the IOF arena. We calculated the percentage of time subjects spent in proximity to the grate during the father condition by dividing the amount of time in proximity to the father by 300 seconds. We assumed greater increase in percentage of time spent in close proximity to the father during testing indicated a higher expression of bond-related behaviors.

IOF Grate

We also quantified the amount of time the females spent physically touching the grate in the IOF arena. Females had to have at least one hand or foot on the grate to be considered touching the grate (touching the grate with the tail or a different part of the body did not count as grate touching in this test to mirror how grate touching is scored in preference tests [Carp et al., 2016]). We calculated the percentage of time spent touching the grate during the father condition of IOF testing as an additional measure of infant proximity maintenance. We also interpreted a greater percentage of time spent touching the grate as indicating a higher expression of bond-related behaviors.

Infant Proximity

From the day an infant is born until they are nine months old, we conduct spot checks every two hours, five days per week, to record who is carrying the infant (mother, father, or sibling) or whether the infant is out of contact with any family member and independently moving about the home-cage (Karaskiewicz et al., 2021). The percentage of time spent being carried by a family member out of all checks recorded can give us a measure of the quality of parental care an infant receives. For the present study, we quantified the percentage of time females were carried by their father out of all scan samples collected for that subject. We assume greater percentage of time being carried by the father early in life could result in a higher expression of father-daughter bond-related behaviors.

Pair Affiliation

In addition to the infant scan samples, as soon as a titi monkey pair is established, our lab conducts pair-mate scan samples every two hours to record where pair-mates are in relation to each other (Karaskiewicz et al., 2021). The four behaviors recorded on our check sheets are proximity, contact, tail-twining, and nothing. Check sheets are completed five days per week for the entire time that a pair is together. This measure of pair-mate affiliation gives us an indication of the overall level of affiliation between partners. We assume greater percentage of time in affiliative contact is an indicator of higher expression of pair-bond related behaviors in this species. For the present study, we quantified the percentage of time partners were in proximity, contact, or tail-twining out of all scan samples collected on the partners during the first 6 months of pairing.

Parent Affiliation

We also quantified affiliation for the parents of our subjects when they were infants and juveniles using the same scan samples used to measure *Pair Affiliation*. This measure of pair-mate affiliation gives us an indication of the overall level of affiliation that the infant observes in the natal group. In human studies, parents' bond-related has been found to be a predictor of children's future romantic bond-related (Gager et al., 2016); therefore, this measure may help us better understand variation in titi monkey attachment. We assume greater percentage of time in affiliative contact is an indicator of higher expression of pair-bond related behavior in this species, and may set the foundation for infants to form stronger bonds as adults. For the present study, we quantified the percentage of time parents were in proximity, contact, or tail-twining out of all scan samples collected on the parents during the first 14 months of our subjects' lives.

Statistical Analyses: Experiment 1

For Experiment 1, our models were specified as: $y_{ij} = \beta_0 + \beta_1 x^{(1)}_{ij} + \cdots + \beta_m x^{(m)}_{ij} + \eta_{i0} + e_{ij}$. In this model, y_{ij} denotes the *j*th observation taken of individual *i* for outcome variable *y*. Individual covariates included in the model are represented by *x* (covariates 1-m), which have fixed effects common to all individuals in the population (β). This model also includes a random effect of ID that is specific to each individual (η_{i0}). Residual errors are represented by e_{ij}. We used a log likelihood ratio test to compare model fit to determine whether removing any non-significant fixed effects resulted in a better fitting model (Supplementary Table S6). The one final model represented the most likely hypothesized relationship between parameters given the data. We acknowledge this final model can be data sensitive and not guaranteed to replicate to other data sets. We used this model as a plausible representation of the data but recognize that other representations are likely. We performed a sensitivity analysis using G*Power 3 prior to the

main analysis to determine the minimum effect size (Cohen's f^2) that we could reliably interpret in each model. We only interpreted results for predictors that had an alpha of $\leq$ .05 and an effect size larger than those determined to be reliable by our sensitivity analysis.

We created a Zone Ratio score by multiplying the time females spent in their partner's preference zone by +1, the time in the father's preference zone by -1, and the time in the neutral zone by 0, and summing these three values per observation. Positive values represented more time in the partner's preference zone while negative values represented more time in the father's preference zone. Values closer to zero either indicated a lack of choice between the father and partner, with the female spending relatively the same amount of time in each zone, or a preference for the non-social areas of the testing arena.

The residuals for Zone Ratio scores were normally distributed; therefore, no transformations were needed. Based on stepwise regression analyses, the variables *Juvenile Parent Preference, Infant Proximity, IOF Grate, IOF Locomotion, IOF Vocalizations, Juvenile Vocalizations, and Juvenile Locomotion* significantly predicted Zone Ratio scores. We then compared a series of general linear mixed effects models with different combinations of these variables with *Test Number, Observation Number,* and *Partner Experience* (experienced vs naïve). ID was included as a random, repeated measure. *Test Number* was allowed to interact with all bond-related-related behaviors. The best-fitting model, based on a log likelihood ratio test, only included the main effects of *Test Number* and *Juvenile Parent Preference* (Supplementary Table S6). The minimum effect size that we could reliably interpret in this model was f^2 = 0.316.

We were also interested in understanding what factors explain variability in the amount of time females spent within a particular preference zone; therefore, we examined duration of time

in each zone separately. The residuals for partner preference zone were normally distributed following a square-root transformation. Based on stepwise regression analyses, the variables *Juvenile Proximity, Juvenile Parent Preference,* and *Juvenile Parent Choice* significantly predicted partner preference zone duration. We then compared a series of general linear mixed-effects models with different combinations of these variables with *Test Number, Observation Number,* and *Partner Experience* (experienced vs naïve). ID was included as a random, repeated measure. *Test Number* was allowed to interact with all bond-related-related behaviors. The best-fitting model, based on a log likelihood ratio test, only included the main effects of test number, *Juvenile Proximity, Juvenile Parent Preference,* and *Juvenile Parent Choice* (Supplementary Table S6). The minimum effect size that we could reliably interpret in this model was $f^2 = 0.318$.

Residuals for father preference zone duration were normally distributed following a square-root transformation. Based on stepwise regression analyses, the variables *Juvenile Parent Preference,* and *Juvenile Locomotion* significantly predicted father preference zone duration. We then compared a series of general linear mixed-effects models with different combinations of these variables with *Test Number, Observation Number, and Partner Experience* (experienced vs naïve). ID was included as a random, repeated measure. *Test Number* was allowed to interact with all bond-related-related behaviors. The best-fitting model, based on a log likelihood ratio test, only included the main effects of *Observation Number* and *Juvenile Parent Preference* (Supplementary Table S6). The minimum effect size that we could reliably interpret in this model was $f^2 = 0.316$.

When females were within the partner or father preference zones, they could interact at the grate, which allowed the closest access to the stimulus animal possible. We wanted to know what variables predicted differences in grate-touching behavior in our subjects. The residuals for

partner grate touch duration were not normally distributed so we used a cube-root transformation. Based on stepwise regression analyses, the variables *Juvenile Proximity, Juvenile Parent Preference, IOF Locomotion, Juvenile Vocalization, and Pair Affiliation* significantly predicted partner grate touch duration. We then compared a series of general linear mixed-effects models with different combinations of these variables with *Test Number, Observation Number,* and *Partner Experience* (experienced vs naïve). ID was included as a random, repeated measure. *Test Number* was allowed to interact with all bond-related-related behaviors. The best-fitting model, based on a log likelihood ratio test, included *Test Number,* the main effects of all tested bond-related variables, and an interaction between *Test Number* and *Juvenile Parent Preference* (Supplementary Table S6). The minimum effect size that we could reliably interpret in this model was $f^2 = 0.323$.

The residuals for father grate touch duration were not normally distributed so we used a cube-root transformation. Based on stepwise regression analyses, the variables *Infant Proximity, IOF Proximity, IOF Vocalizations, Juvenile Vocalizations, Juvenile Locomotion,* and *Parent Affiliation* significantly predicted father grate touch duration. We then compared a series of general linear mixed-effects models with different combinations of these variables with *Test Number, Observation Number,* and *Partner Experience* (experienced vs naïve). ID was included as a random, repeated measure. *Test Number* was allowed to interact with all bond-related-related behaviors. The best-fitting model, based on a log likelihood ratio test, included *Test Number, Observation Number,* all main effects of bond-related variables, and interaction effects between *Test Number* and *IOF Proximity, IOF Vocalization,* and *Parent Affiliation* (Supplementary Table S6). The minimum effect size that we could reliably interpret in this model was $f^2 = 0.333$.

The Zone Ratio score does not distinguish between a female spending a lot of time in other and a female spending equal time in partner and father zones. To disentangle these two outcomes, we created a Social Zone score, which is the sum of time in the partner's preference zone plus the time in the father's zone. Larger values represent a greater amount of time in a social zone, which would indicate a lack of choice between the father and partner if Zone Ratio is close to zero. Smaller values represent more time in the non-social areas of the testing arena. Across all three tests females spent the majority of the 1800 possible seconds in one of the two social preference zones (Mean = 1255.57, SD = 279.52 seconds), and exhibited a significant increase in time in the father and partner preference zones from one-week pre-pairing to six-months post-pairing (Mean = 1340.38, SD = 227.56 seconds).

The residuals for Social Zone were normally distributed; therefore, no transformation was necessary. Based on stepwise regression analyses, the variables *Juvenile Parent Preference, Juvenile Parent Choice, Infant Proximity, IOF Grate, IOF Locomotion, IOF Vocalizations,* and *Juvenile Vocalizations* significantly predicted Social Zone scores. We then compared a series of general linear mixed-effects models with different combinations of these variables with *Test Number, Observation Number,* and *Partner Experience* (experienced vs naïve). ID was included as a random, repeated measure. *Test Number* was allowed to interact with all bond-related behaviors. The best-fitting model, based on a log likelihood ratio test, did not include the main effects of *Observation Number* and *Partner Experience*; however, it did include all other main and interaction effects (Supplementary Table S6).

Any time females spent outside of the partner or father's preference zone was considered time spent in "other". We wanted to understand what factors explained variability in the amount of time females spent in the non-social parts of the testing arena. The residuals for other

preference zone duration were not normally distributed so we used a square-root transformation. Based on stepwise regression analyses, the variables *Juvenile Parent Preference, Infant Proximity, IOF Grate, IOF Locomotion, IOF Vocalizations, Juvenile Vocalizations,* and *Juvenile Locomotion* significantly predicted other preference zone duration. We then compared a series of general linear mixed-effects models with different combinations of these variables with *Test Number, Observation Number,* and *Partner Experience* (experienced vs naïve). ID was included as a random, repeated measure. *Test Number* was allowed to interact with all bond-related-related behaviors. The best-fitting model, based on a log likelihood ratio test, included the main effects of *Test Number* and all tested bond-related variables with no interaction effects (Supplementary Table S6).

Statistical Analyses: Experiment 2

For Experiment 2, our models were specified as: $y_{ij} = \beta_0 + \beta_1 x^{(1)}_{ij} + \cdots + \beta_m x^{(m)}_{ij} + \eta_{i0} + e_{ij}$. In this model, y_{ij} denotes the *j*th observation taken of individual *i* for outcome variable *y.* Individual covariates included in the model are represented by *x* (covariates 1-m), which have fixed effects common to all individuals in the population (β). This model also includes a random effect of ID that is specific to each individual (η_{i0}). Residual errors are represented by e_{ij}. We performed a sensitivity analysis using G*Power 3 prior to the main analysis to determine the minimum effect size (Cohen's f^2) that we could reliably interpret for each model. We only interpreted results for predictors that had an alpha of $\leq .05$ and an effect size larger than those determined to be reliable by our sensitivity analysis.

A log transformation using base e ($\log_e$ transformation) resulted in normal distribution of the residuals for whole brain activity. Based on stepwise regression analyses, the variables *Juvenile Proximity*, *Juvenile Parent Choice, IOF Proximity,* and *IOF Locomotion* significantly

predicted whole brain activity. We then compared a series of general linear mixed effects models with different combinations of these variables with *Condition* (father, separated from father, partner, separated from partner) and *Side* (left, right). ID was included as a random, repeated measure. *Condition* was allowed to interact with all bond-related-related behaviors. The best-fitting model, based on a log likelihood ratio test, included all main and interaction effects except for *Side* (Supplementary Table S7). The minimum effect size that we could reliably interpret in this model was $f^2 = 0.235$.

Residuals were normally distributed for social salience network activity following a log-transformation. Based on stepwise regression analyses, the variables *Juvenile Proximity* and *Parent Affiliation* significantly predicted social salience network activity. We then compared a series of general linear mixed effects models with different combinations of these variables with *Condition* (father, separated from father, partner, separated from partner), *Region* (amygdala, hypothalamus, lateral septum, nucleus accumbens, ventral pallidum, ventral tegmental area), and *Side* (left, right). ID was included as a random, repeated measure. *Condition* was allowed to interact with all bond-related-related behaviors. The best-fitting model, based on a log likelihood ratio test, included all main and interaction effects except for *Side* (Supplementary Table S7). The minimum effect size that we could reliably interpret in this model was $f^2 = 0.233$.

We used a $\log_e$ transformation to normalize the distribution of the residuals for cerebellum activity. Based on stepwise regression analyses, the variables *Juvenile Proximity* and *Parent Affiliation* significantly predicted cerebellum activity. We then compared a series of general linear mixed effects models with different combinations of these variables with *Condition* (father, separated from father, partner, separated from partner). ID was included as a random, repeated measure. *Condition* was allowed to interact with all bond-related-related

behaviors. The best-fitting model, based on a log likelihood ratio test, included all main effects and an interaction between *Condition* and *Juvenile Proximity* (Supplementary Table S7). The minimum effect size that we could reliably interpret in this model was $f^2 = 0.232$.

The residuals for periaqueductal gray activity were normally distributed following a $\log_e$ transformation. Based on stepwise regression analyses, the variables *Juvenile Proximity, Juvenile Parent Choice, IOF Proximity, IOF Grate, Juvenile Vocalization,* and *Parent Affiliation* significantly predicted PAG activity. We then compared a series of general linear mixed effects models with different combinations of these variables with *Condition* (father, separated from father, partner, separated from partner) and *Side* (left, right). ID was included as a random, repeated measure. *Condition* was allowed to interact with all bond-related-related behaviors. The best-fitting model, based on a log likelihood ratio test, included all main effects except for *Side* in addition to interactions between *Condition* and *Juvenile Proximity, IOF Grate, Juvenile Vocalization,* and *Parent Affiliation* (Supplementary Table S7). The minimum effect size that we could reliably interpret in this model was $f^2 = 0.237$.

The distribution of the residuals for plasma cortisol was normal, so no transformation was needed. Based on stepwise regression analyses, the variables *Juvenile Parent Preference* and *Infant Proximity* significantly predicted cortisol activity. We then compared a series of general linear mixed effects models with different combinations of these variables with *Condition* (father, separated from father, partner, separated from partner). ID was included as a random, repeated measure. *Condition* was allowed to interact with all bond-related-related behaviors. The best-fitting model, based on a log likelihood ratio test, included all main effects and the interaction between *Condition* and *Infant Proximity* (Supplementary Table S7). The minimum effect size that we could reliably interpret in this model was $f^2 = 0.232$.

SUPPLEMENTARY TABLES

Supplementary Table S1. Results from Experiment 1 general linear mixed-effects models. IOFLine = *IOF Locomotion;* IOFVoc = *IOF Vocalization;* JuvVoc = *Juvenile Vocalization;* JuvLoc = *Juvenile Locomotion;* JuvReun = *Juvenile Proximity;* PropPar = *Juvenile Parent Preference;* CnRPar = *Juvenile Choice;* InfCarry = *Infant Proximity;* IOFProx = *IOF Proximity;* IOFGrate = *IOF Grate;* ParAff = *Parent Affiliation;* PairAff = *Pair Affiliation*

S1a. Zone Ratio

	β	SE	95% CI	t-value	p
(Intercept)	-270.422	84.793	[-435.305, -105.540]	-3.189	0.002
TestNo2	59.511	119.916	[-173.668, 292.690]	0.496	0.8732
TestNo3	328.889	119.916	[95.710, 562.068]	2.743	0.019
PropPar	-7.780	2.278	[-12.210, -3.350]	-3.415	< .001

S1b. Partner Zone

	β	SE	95% CI	t-value	p
(Intercept)	20.552	0.960	[18.700, 22.404]	21.412	< .001
TestNo2	0.521	1.357	[-2.098, 3.141]	0.384	0.922
TestNo3	5.196	1.357	[2.576, 7.815]	3.828	< .001
JuvReun	0.060	0.021	[0.019, 0.101]	2.853	0.005
PropPar	-0.155	0.030	[-0.212, -0.097]	-5.167	< .001
CnRPar	0.101	0.036	[0.032, 0.171]	2.806	0.006

S1c. Father Zone

	β	SE	95% CI	t-value	p
(Intercept)	28.091	1.317	[25.571, 30.611]	21.323	< .001
ObsNo	-0.811	0.373	[-1.545, -0.077]	-2.173	0.032
PropPar	0.055	0.032	[-0.007, 0.118]	1.710	0.131

S1d. Partner Grate Touch

	β	SE	95% CI	t-value	p
(Intercept)	4.058	0.192	[3.742, 4.374]	21.178	< .001
TestNo2	-0.280	0.232	[-0.727, 0.166]	-1.208	0.4506
TestNo3	0.120	0.232	[-0.327, 0.566]	0.515	0.8641
JuvReun	0.019	0.010	[0.005, 0.033]	1.823	0.166
PropPar	-0.012	0.011	[-0.029, 0.004]	-1.157	0.285
IOFLine	-0.001	0.001	[-0.003, 0.000]	-1.085	0.357
JuvVoc	0.006	0.004	[0.001, 0.011]	1.482	0.235
PairAff	-0.034	0.025	[-0.068, 0.000]	-1.338	0.273
TestNo2:PropPar	-0.037	0.011	[-0.058, -0.016]	-3.401	< .001
TestNo3:PropPar	0.014	0.011	[-0.007, 0.035]	1.289	0.200

S1e. Father Grate Touch

	β	SE	95% CI	t-value	p
(Intercept)	5.554	0.301	[5.078, 6.031]	18.431	< .001
TestNo2	-0.271	0.223	[-0.695, 0.154]	-1.213	0.4478
TestNo3	-0.435	0.223	[-0.859, -0.010]	-1.949	0.1298
ObsNo	-0.260	0.064	[-0.383, -0.138]	-4.043	< .001
InfCarry	0.112	0.070	[0.039, 0.185]	1.587	0.253
IOFProx	0.005	0.011	[-0.008, 0.019]	0.494	0.650
IOFVoc	-0.008	0.007	[-0.015, 0.000]	-1.163	0.346
JuvVoc	0.004	0.004	[0.000, 0.008]	0.942	0.446
JuvLoc	-0.001	0.001	[-0.002, 0.000]	-1.509	0.270
ParAff	-0.067	0.044	[-0.120, -0.013]	-1.501	0.231
TestNo2:IOFProx	-0.006	0.009	[-0.024, 0.011]	-0.695	0.489
TestNo3:IOFProx	0.030	0.009	[0.012, 0.047]	3.184	0.002
TestNo2:IOFVoc	0.008	0.004	[0.001, 0.015]	2.156	0.033
TestNo3:IOFVoc	-0.003	0.004	[-0.009, 0.004]	-0.692	0.490
TestNo2:ParAff	0.039	0.033	[-0.024, 0.101]	1.182	0.240
TestNo3:ParAff	-0.022	0.033	[-0.084, 0.040]	-0.683	0.496

S1f. Social Zone

	β	SE	95% CI	t-value	p
(Intercept)	1197.6222	35.3743	[1134.304, 1260.940]	33.856	< .001
TestNo2	31.0889	50.0268	[-58.456, 120.634]	0.621	0.536
TestNo3	142.7556	50.0268	[53.210, 232.301]	2.854	0.005
PropPar	7.3395	4.1635	[-0.113, 14.792]	1.763	0.081
CnRPar	-1.4806	4.3246	[-9.221, 6.260]	-0.342	0.733
InfCarry	5.5344	19.2642	[-28.947, 40.016]	0.287	0.774
IOFGrate	-0.4018	4.9142	[-9.198, 8.394]	-0.082	0.935
IOFLine	-0.4757	0.4508	[-1.283, 0.331]	-1.055	0.294
IOFVoc	-3.208	1.7225	[-6.291, -0.125]	-1.862	0.065
JuvVoc	4.1589	1.4992	[1.475, 6.842]	2.774	0.007
TestNo2:PropPar	1.0576	5.8881	[-9.482, 11.597]	0.18	0.858
TestNo3:PropPar	-4.2803	5.8881	[-14.820, 6.259]	-0.727	0.469
TestNo2:CnRPar	-5.9018	6.1159	[-16.849, 5.045]	-0.965	0.337
TestNo3:CnRPar	-1.5553	6.1159	[-12.502, 9.392]	-0.254	0.800
TestNo2:InfCarry	-55.4829	27.2437	[-104.248, -6.718]	-2.037	0.044
TestNo3:InfCarry	-10.6281	27.2437	[-59.393, 38.137]	-0.39	0.697
TestNo2:IOFGrate	-9.0119	6.9498	[-21.452, 3.428]	-1.297	0.197
TestNo3:IOFGrate	2.275	6.9498	[-10.165, 14.715]	0.327	0.744
TestNo2:IOFLine	-0.2899	0.6376	[-1.431, 0.851]	-0.455	0.650
TestNo3:IOFLine	0.1563	0.6376	[-0.985, 1.298]	0.245	0.807
TestNo2:IOFVoc	3.3193	2.4359	[-1.041, 7.679]	1.363	0.176
TestNo3:IOFVoc	2.4053	2.4359	[-1.955, 6.766]	0.987	0.326
TestNo2:JuvVoc	-1.5149	2.1202	[-5.310, 2.280]	-0.715	0.476
TestNo3:JuvVoc	-3.5362	2.1202	[-7.331, 0.259]	-1.668	0.098

S1g. Other Zone

	β	SE	95% CI	t-value	p
(Intercept)	23.670	0.837	[22.079, 25.260]	28.268	<2e-16
TestNo2	-0.907	1.184	[-3.156, 1.343]	-0.766	0.445
TestNo3	-2.952	1.184	[-5.201, -0.703]	-2.493	0.014

PropPar	-0.101	0.044	[-0.184, -0.018]	-2.319	0.022
InfCarry	0.082	0.229	[-0.352, 0.517]	0.360	0.720
IOFGrate	0.008	0.051	[-0.088, 0.105]	0.160	0.873
IOFLine	0.010	0.006	[-0.002, 0.022]	1.571	0.119
IOFVoc	0.040	0.027	[-0.011, 0.091]	1.474	0.143
JuvVoc	-0.047	0.020	[-0.086, -0.008]	-2.281	0.024
JuvLoc	0.003	0.002	[0.000, 0.007]	1.749	0.083

Supplementary Table S2. Results from Experiment 2 general linear mixed-effects models. Conditionpartner = tested with partner six-months post-pairing; ConditionsepF = separated from father while still living in natal group; ConditionsepP = separated from partner six-months post-pairing; IOFLine = *IOF Locomotion;* IOFVoc = *IOF Vocalization;* JuvVoc = *Juvenile Vocalization;* JuvLoc = *Juvenile Locomotion;* JuvReun = *Juvenile Proximity;* PropPar = *Juvenile Parent Preference;* CnRPar = *Juvenile Choice;* InfCarry = *Infant Proximity;* IOFProx = *IOF Proximity;* IOFGrate = *IOF Grate;* ParAff = *Parent Affiliation;* PairAff = *Pair Affiliation*

S2a. Social Salience Network

	β	SE	95% CI	t-value	p
(Intercept)	7.534	0.054	[7.437, 7.631]	138.837	< .001
Conditionpartner	-0.365	0.034	[-0.430, -0.300]	-10.855	< .001
ConditionsepF	-0.074	0.034	[-0.139, -0.009]	-2.210	0.122
ConditionsepP	-0.421	0.034	[-0.486, -0.356]	-12.494	< .001
RegionHYP	-1.689	0.041	[-1.769, -1.610]	-40.972	< .001
RegionLS	-2.998	0.041	[-3.078, -2.918]	-72.710	< .001
RegionNAC	-0.981	0.041	[-1.060, -0.901]	-23.783	< .001
RegionVP	-4.025	0.041	[-4.105, -3.946]	-97.634	< .001
RegionVTA	-3.268	0.041	[-3.347, -3.188]	-79.252	< .001
JuvReun	0.000	0.002	[-0.003, 0.002]	-0.271	0.793
ParAff	0.009	0.006	[-0.003, 0.021]	1.382	0.200
Conditionpartner:JuvReun	-0.006	0.001	[-0.008, -0.003]	-4.854	< .001
ConditionsepF:JuvReun	-0.012	0.001	[-0.014, -0.010]	-10.218	< .001
ConditionsepP:JuvReun	-0.002	0.001	[-0.004, 0.000]	-1.537	0.125
Conditionpartner:ParAff	0.022	0.005	[0.013, 0.031]	4.787	< .001
ConditionsepF:ParAff	-0.007	0.005	[-0.016, 0.002]	-1.501	0.134
ConditionsepP:ParAff	0.017	0.005	[0.008, 0.026]	3.718	< .001

S2b. Periaqueductal Gray (PAG)

	β	SE	95% CI	t-value	p
(Intercept)	5.491	0.056	[5.416, 5.567]	97.325	< .001
Conditionpartner	-0.363	0.060	[-0.467, -0.259]	-6.071	< .001
ConditionsepF	-0.083	0.060	[-0.186, 0.021]	-1.382	0.516
ConditionsepP	-0.442	0.060	[-0.546, -0.339]	-7.403	< .001
JuvReun	-0.007	0.003	[-0.010, -0.003]	-2.560	0.051
CnRPar	-0.003	0.003	[-0.006, 0.000]	-1.119	0.379
IOFProx	0.004	0.002	[0.001, 0.006]	1.558	0.259
IOFGrate	-0.016	0.006	[-0.023, -0.009]	-2.697	0.062
JuvVoc	-0.006	0.001	[-0.008, -0.004]	-3.985	0.008
ParAff	-0.023	0.012	[-0.038, -0.008]	-1.965	0.116
Conditionpartner:JuvReun	0.001	0.003	[-0.003, 0.006]	0.527	0.601
ConditionsepF:JuvReun	-0.009	0.003	[-0.013, -0.004]	-3.207	0.002
ConditionsepP:JuvReun	0.003	0.003	[-0.002, 0.008]	1.096	0.278
Conditionpartner:IOFGrate	0.010	0.005	[0.002, 0.018]	2.090	0.042
ConditionsepF:IOFGrate	0.004	0.005	[-0.004, 0.012]	0.842	0.404
ConditionsepP:IOFGrate	0.023	0.005	[0.014, 0.031]	4.757	< .001
Conditionpartner:JuvVoc	0.006	0.002	[0.004, 0.009]	4.244	< .001
ConditionsepF:JuvVoc	0.003	0.002	[0.001, 0.006]	2.203	0.032
ConditionsepP:JuvVoc	0.006	0.002	[0.003, 0.009]	3.938	< .001
Conditionpartner:ParAff	0.044	0.011	[0.025, 0.063]	4.082	< .001
ConditionsepF:ParAff	0.005	0.011	[-0.013, 0.024]	0.495	0.623
ConditionsepP:ParAff	0.054	0.011	[0.036, 0.073]	5.058	< .001

S2c. Cerebellum

	β	SE	95% CI	t-value	p
(Intercept)	10.705	0.091	[10.546, 10.865]	117.090	< .001
Conditionpartner	-0.370	0.129	[-0.281, 0.170]	-2.858	0.0432
ConditionsepF	-0.056	0.129	[-0.595, -0.144]	-0.429	0.9728
ConditionsepP	-0.455	0.129	[-0.681, -0.230]	-3.520	0.0101
JuvReun	-0.001	0.003	[-0.006, 0.004]	-0.333	0.742

ParAff	0.012	0.006	[0.002, 0.023]	1.992	0.057
Conditionpartner:JuvReun	-0.005	0.004	[-0.020, -0.005]	-1.089	0.286
ConditionsepF:JuvReun	-0.012	0.004	[-0.013, 0.003]	-2.770	0.010
ConditionsepP:JuvReun	-0.001	0.004	[-0.009, 0.006]	-0.296	0.769

S2d. Whole Brain

	β	SE	95% CI	t-value	p
(Intercept)	12.633	0.054	[12.544, 12.722]	235.496	< .001
Conditionpartner	-0.378	0.074	[-0.504, -0.252]	-5.098	< .001
ConditionsepF	-0.050	0.074	[-0.176, 0.076]	-0.676	0.906
ConditionsepP	-0.411	0.074	[-0.537, -0.285]	-5.540	< .001
JuvReun	-0.003	0.002	[-0.007, 0.001]	-1.385	0.176
CnRPar	-0.002	0.003	[-0.007, 0.003]	-0.523	0.604
IOFProx	0.005	0.003	[0.001, 0.009]	1.916	0.064
IOFLine	-0.001	0.000	[-0.001, 0.000]	-1.244	0.223
Conditionpartner:JuvReun	-0.007	0.003	[-0.012, -0.002]	-2.354	0.023
ConditionsepF:JuvReun	-0.014	0.003	[-0.019, -0.008]	-4.484	< .001
ConditionsepP:JuvReun	-0.002	0.003	[-0.007, 0.003]	-0.786	0.436
Conditionpartner:CnRPar	0.001	0.004	[-0.006, 0.008]	0.282	0.780
ConditionsepF:CnRPar	0.001	0.004	[-0.006, 0.008]	0.261	0.795
ConditionsepP:CnRPar	-0.010	0.004	[-0.017, -0.002]	-2.267	0.028
Conditionpartner:IOFProx	0.001	0.004	[-0.005, 0.007]	0.363	0.718
ConditionsepF:IOFProx	-0.004	0.004	[-0.010, 0.002]	-1.168	0.249
ConditionsepP:IOFProx	0.003	0.004	[-0.003, 0.009]	0.861	0.393
Conditionpartner:IOFLine	-0.001	0.001	[-0.002, 0.000]	-1.521	0.135
ConditionsepF:IOFLine	0.000	0.001	[-0.001, 0.001]	-0.181	0.857
ConditionsepP:IOFLine	0.000	0.001	[-0.001, 0.001]	0.414	0.681

S2e. Cortisol (CORT)

	β	SE	95% CI	t-value	p
(Intercept)	797.004	48.014	[714.155, 879.854]	16.599	< .001
ConditionsepF	15.593	52.710	[-78.855, 110.042]	0.296	0.991

Conditionpartner	38.902	52.710	[-55.547, 133.351]	0.738	0.881
ConditionsepP	125.364	52.710	[30.916, 219.813]	2.378	0.112
PropPar	-3.494	1.817	[-6.741, -0.247]	-1.923	0.103
InfCarry	43.829	9.642	[27.173, 60.486]	4.546	< .001
ConditionsepF:InfCarry	-11.974	10.033	[-29.953, 6.004]	-1.193	0.246
Conditionpartner:InfCarry	-20.154	10.033	[-38.132, -2.175]	-2.009	0.058
ConditionsepP:InfCarry	-25.867	10.033	[-43.846, -7.889]	-2.578	0.018

Supplementary Table S3. Ethogram for partner-father preference test outcome variables

Behavior	Definition
Zone Ratio	Ratio indicating which zone (partner, father, other) subject spends the most time in. Calculated by multiplying time in partner zone by +1, time in father zone by -1, time in other zone by 0, and summing those three values
Partner Zone	Total time subject is located in the preference zone next to the partner
Father Zone	Total time subject is located in the preference zone next to the father pair
Partner Grate	Total time subject touches the grated window between the test cage and their partner's cage using hands or feet
Father Grate	Total time subject touches the grated window between the test cage and the father's cage using hands or feet
Social Zone	Total test time subject is located in either the partner or the father zone.
Other Zone	Total time subject is located in any location in the test cage other than the partner or father preference zones

Supplementary Table S4 Ethogram for quantification of bond-related behaviors

Behavior	Definition
Separation Distress Behaviors	
IOF Locomotion	Percent change in line crossing from the father to the empty box condition of infant open field (IOF) testing
IOF Vocalization	Percent change in vocalizations emitted from the father to the empty box condition of IOF testing
Juvenile Locomotion	Percent change in locomotion behavior from the stress buffered to the separation distress condition of juvenile separation testing when subjects are treated with saline
Juvenile Vocalization	Percent change in total vocalizations emitted from the stress buffered to the separation distress condition of juvenile separation testing when subjects are treated with saline
Proximity Maintenance Behaviors	
Juvenile Proximity	Percentage of time subject spends in proximity, contact, or tail-twining with the father during a 15-minute reunion period immediately following juvenile social separation testing when subjects are treated with saline
Juvenile Parent Preference	Percentage of time subject spends in the parent proximity zone of juvenile parent preference tests when subjects are treated with saline
Juvenile Parent Choice	Percentage of times subject chooses parents during the catch and release testing immediately following juvenile parent preference tests when subjects are treated with saline
IOF Proximity	Percentage of time subject spends in proximity to the father during the father condition of IOF testing

IOF Grate	Percentage of time subject spends touching the grate in the IOF arena during the father condition of IOF testing
Infant Proximity	Percentage of time subject was carried by the father during the first nine months of life as measured during daily scan samples
Affiliation Behaviors	
Pair Affiliation	Percentage of time subject spent in proximity (within ~6 inches of one another), contact (physical body contact), or tail-twining (sitting with tails wrapped together) with their partner during the first six months of pairing as measured during daily scan samples
Parent Affiliation	Percentage of time parents of subjects spent in proximity (within ~6 inches of one another), contact (physical body contact), or tail-twining (sitting with tails wrapped together) with each other during the first 14 months of the subject's life as measured during daily scan samples

Supplementary Table S5. Pearson correlation matrix of bond-related variables. Significant correlations (p < .05) indicated in bold. IOFLine = *IOF Locomotion;* IOFVoc = *IOF Vocalization;* JuvVoc = *Juvenile Vocalization;* JuvLoc = *Juvenile Locomotion;* JuvReun = *Juvenile Proximity;* PropPar = *Juvenile Parent Preference;* CnRPar = *Juvenile Choice;* InfCarry = *Infant Proximity;* IOFProx = *IOF Proximity;* IOFGrate = *IOF Grate;* ParAff = *Parent Affiliation;* PairAff = *Pair Affiliation*

	IOFLine	IOFVoc	JuvVoc	JuvLoc	JuvReun	PropPar	CnRPar	InfCarry	IOFProx	IOFGrate	ParAff	PairAff
IOFLine	1.00											
IOFVoc	**0.69**	1.00										
JuvVoc	**0.52**	**0.42**	1.00									
JuvLoc	**-0.20**	**-0.25**	**-0.35**	1.00								
JuvReun	**-0.49**	**-0.44**	**-0.62**	**0.20**	1.00							
PropPar	0.10	**0.44**	**-0.37**	**0.23**	0.09	1.00						
CnRPar	**0.27**	**0.20**	**0.29**	**0.40**	**-0.34**	**0.44**	1.00					
InfCarry	0.00	**0.56**	-0.13	0.14	-0.01	**0.41**	**-0.19**	1.00				
IOFProx	**0.49**	0.11	-0.05	**-0.22**	**-0.26**	**0.31**	0.07	**-0.22**	1.00			
IOFGrate	0.13	**-0.19**	**-0.36**	-0.11	**0.25**	-0.06	**-0.36**	**-0.46**	**0.42**	1.00		
ParAff	**-0.52**	**-0.34**	**-0.26**	**-0.31**	0.02	-0.01	**-0.18**	0.09	0.04	**-0.44**	1.00	
PairAff	0.08	0.09	0.15	**-0.47**	**0.49**	**-0.38**	**-0.47**	-0.05	**-0.38**	0.14	**-0.18**	1.00

Supplementary Table S6. Experiment 1 Log likelihood ratio test results comparing model fit for general linear mixed-effects models.

Supplementary 6. Zone Ratio

	npar	AIC	BIC	logLik	deviance	Chisq	Df	Pr(>Chisq)
ZoneRatioonlyTestNo	5	2110.5	2125.1	-1050.3	2100.5			
ZoneRatioonlysig	6	2103.8	2121.2	-1045.9	2091.8	8.7101	1	0.003164
ZoneRationoInt	8	2104.4	2127.6	-1044.2	2088.4	3.435	2	0.179517
ZoneRationoPartEXP	14	2111.4	2152	-1041.7	2083.4	5.0173	6	0.541598
ZoneRationoObs	15	2113.3	2156.9	-1041.7	2083.3	0.0266	1	0.870528
ZoneRatioAll	16	2112.8	2159.3	-1040.4	2080.8	2.5363	1	0.111257
	npar	AIC	BIC	logLik	deviance	Chisq	Df	Pr(>Chisq)
Null1	3	2114.9	2123.6	-1054.5	2108.9			
ZoneRatioonlysig	6	2103.8	2121.2	-1045.9	2091.8	17.093	3	0.0006764

Supplementary 6. Partner Zone

	npar	AIC	BIC	logLik	deviance	Chisq	Df	Pr(>Chisq)
PPZonlyTestNo	5	907.95	922.47	-448.97	897.95			
PPZnoInt	8	895.81	919.05	-439.9	879.81	18.1428	3	0.000411
PPZnoPartEXP	14	897.22	937.89	-434.61	869.22	10.5902	6	0.101898
PPZnoObs	15	898.87	942.45	-434.43	868.87	0.3492	1	0.554564
PPZAll	16	900.28	946.77	-434.14	868.28	0.5813	1	0.445798
	npar	AIC	BIC	logLik	deviance	Chisq	Df	Pr(>Chisq)
Null1	3	920.46	929.18	-457.23	914.46			
PPZnoInt	8	895.81	919.05	-439.9	879.81	34.657	5	1.76E-06

Supplementary 6. Father Zone

	npar	AIC	BIC	logLik	deviance	Chisq	Df	Pr(>Chisq)
FPZonlysig	5	884.36	898.89	-437.18	874.36			
FPZonlyTestNo	5	890.35	904.88	-440.18	880.35	0	0	
FPZnoInt	8	885.56	908.81	-434.78	869.56	10.7903	3	0.01292
FPZnoPartEXP	12	886.64	921.51	-431.32	862.64	6.9211	4	0.14011
FPZAll	13	887.97	925.74	-430.98	861.97	0.6749	1	0.41136
	npar	AIC	BIC	logLik	deviance	Chisq	Df	Pr(>Chisq)
Null1	3	888.18	896.9	-441.09	882.18			
FPZonlysig	5	884.36	898.89	-437.18	874.36	7.8152	2	0.02009

Supplementary 6. Partner Grate Touch

	npar	AIC	BIC	logLik	deviance	Chisq	Df	Pr(>Chisq)
PGTDonlyTestNo	5	451.49	466.01	-220.74	441.49			
PGTDonlyPropPar	8	430.72	453.97	-207.36	414.72	26.7639	3	6.60E-06
PGTDoneInt	12	426.22	461.09	-201.11	402.22	12.5014	4	0.01399
PGTDnoPartEXP	20	435.35	493.45	-197.67	395.35	6.8744	8	0.55024
PGTDnoObs	21	437.24	498.25	-197.62	395.24	0.1088	1	0.7415
PGTDAll	22	438.7	502.62	-197.35	394.7	0.5395	1	0.46265
	npar	AIC	BIC	logLik	deviance	Chisq	Df	Pr(>Chisq)
Null1	3	450.17	458.89	-222.09	444.17			
PGTDoneInt	12	426.22	461.09	-201.11	402.22	41.948	9	3.36E-06

Supplementary 6 Father Grate Touch

	npar	AIC	BIC	logLik	deviance	Chisq	Df	Pr(>Chisq)
FGTDonlyObsNo	4	438.24	449.86	-215.12	430.24			
FGTDonlyTestNo	5	450.61	465.14	-220.31	440.61	0	1	1
FGTDnoRel	6	438.83	456.27	-213.42	426.83	13.778	1	0.0002057
FGTDonlySig	12	426.43	461.29	-201.22	402.43	24.4056	6	0.0004397
FGTDsigInt	18	424.86	477.16	-194.43	388.86	13.5683	6	0.0348487
FGTDnoPartEXP	24	427.75	497.47	-189.87	379.75	9.1126	6	0.1673444
FGTDAll	25	429.7	502.33	-189.85	379.7	0.0495	1	0.8238596
	npar	AIC	BIC	logLik	deviance	Chisq	Df	Pr(>Chisq)
Null1	3	449.67	458.38	-221.84	443.67			
FGTDsigInt	18	424.86	477.16	-194.43	388.86	54.808	15	1.92E-06

Supplementary 6. Social Zone

	npar	AIC	BIC	logLik	deviance	Chisq	Df	Pr(>Chisq)
PropSocialonlyTestNo	5	1112.8	1127.3	-551.39	1102.8			
PropSocialonlysig	7	1116	1136.3	-551.01	1102	0.7682	2	0.6810726
PropSocialnoInt	12	1104.1	1139	-540.04	1080.1	21.9256	5	0.0005409
PropSocialnoPartEXP	26	1106.4	1182	-527.22	1054.4	25.6457	14	0.0287029
PropSocialnoObs	27	1108.3	1186.8	-527.17	1054.3	0.1074	1	0.7430718
PropSocialAll	28	1106.3	1187.7	-525.17	1050.3	3.9937	1	0.0456715
	npar	AIC	BIC	logLik	deviance	Chisq	Df	Pr(>Chisq)
Null1	3	1116.6	1125.3	-555.29	1110.6			
PropSocialnoPartEXP	26	1106.4	1182	-527.22	1054.4	56.146	23	0.0001351

Supplementary 6. Other Zone

	npar	AIC	BIC	logLik	deviance	Chisq	Df	Pr(>Chisq)
ODonlyTestNo	5	870.15	884.67	-430.07	860.15			
ODonlysig	7	873.5	893.84	-429.75	859.5	0.6437	2	0.7247889
ODnoInt	12	862.69	897.55	-419.35	838.69	20.8114	5	0.0008793
ODnoPartEXPorObsNo	26	870.34	945.87	-409.17	818.34	20.3536	14	0.1193811
ODAll	28	872.14	953.49	-408.07	816.14	2.1919	2	0.3342272
	npar	AIC	BIC	logLik	deviance	Chisq	Df	Pr(>Chisq)
Null1	3	872.55	881.27	-433.28	866.55			
ODnoInt	12	862.69	897.55	-419.35	838.69	27.865	9	0.001005

Supplementary 6 Zone Ratio Models:

ZoneRatioonlyTestNo: ZoneRatio ~ TestNo + (1 | ID)

ZoneRatioonlysig: ZoneRatio ~ TestNo + PropPar + (1 | ID)

ZoneRationoInt: ZoneRatio ~ TestNo + PropPar + CnRPar + JuvVoc + (1 | ID)

ZoneRationoPartEXP: ZoneRatio ~ TestNo + PropPar + CnRPar + JuvVoc + PropPar * TestNo + CnRPar * TestNo + JuvVoc * TestNo + (1 | ID)

ZoneRationoObs: ZoneRatio ~ TestNo + PartnerEXP + PropPar + CnRPar + JuvVoc + PropPar * TestNo + CnRPar * TestNo + JuvVoc * TestNo + (1 | ID)

ZoneRatioAll: ZoneRatio ~ TestNo + ObsNo + PartnerEXP + PropPar + CnRPar + JuvVoc + PropPar * TestNo + CnRPar * TestNo + JuvVoc * TestNo + (1 | ID)

Null1: ZoneRatio ~ 1 + (1 | ID)

Supplementary 6 Partner Preference Zone Models:

PPZonlyTestNo: sqrtPPZD ~ TestNo + (1 | ID)

PPZnoInt: sqrtPPZD ~ TestNo + JuvReun + PropPar + CnRPar + (1 | ID)

PPZnoPartEXP: sqrtPPZD ~ TestNo + JuvReun + PropPar + CnRPar + JuvReun * TestNo + PropPar * TestNo + CnRPar * TestNo + (1 | ID)

PPZnoObs: sqrtPPZD ~ TestNo + PartnerEXP + JuvReun + PropPar + CnRPar + JuvReun * TestNo + PropPar * TestNo + CnRPar * TestNo + (1 | ID)

PPZAll: sqrtPPZD ~ TestNo + ObsNo + PartnerEXP + JuvReun + PropPar + CnRPar + JuvReun * TestNo + PropPar * TestNo + CnRPar * TestNo + (1 | ID)

Null1: sqrtPPZD ~ 1 + (1 | ID)

Supplementary 6 Father Preference Zone Models:

FPZonlysig: sqrtFPZD ~ ObsNo + PropPar + (1 | ID)

FPZonlyTestNo: sqrtFPZD ~ TestNo + (1 | ID)

FPZnoInt: sqrtFPZD ~ TestNo + ObsNo + PropPar + JuvLoc + (1 | ID)

FPZnoPartEXP: sqrtFPZD ~ TestNo + ObsNo + PropPar + JuvLoc + PropPar * TestNo + JuvLoc * TestNo + (1 | ID)

FPZAll: sqrtFPZD ~ TestNo + ObsNo + PartnerEXP + PropPar + JuvLoc + PropPar * TestNo + JuvLoc * TestNo + (1 | ID)

Null1: sqrtFPZD ~ 1 + (1 | ID)

Supplementary 6 Partner Grate Touch Models:

PGTDonlyTestNo: cubePGTD ~ TestNo + (1 | ID)

PGTDonlyPropPar: cubePGTD ~ TestNo + PropPar + PropPar * TestNo + (1 | ID)

PGTDoneInt: cubePGTD ~ TestNo + JuvReun + PropPar + IOFLine + JuvVoc + PairAff + PropPar * TestNo + (1 | ID)

PGTDnoPartEXP: cubePGTD ~ TestNo + JuvReun + PropPar + IOFLine + JuvVoc + PairAff + JuvReun * TestNo + PropPar * TestNo + IOFLine * TestNo + JuvVoc * TestNo + PairAff * TestNo + (1 | ID)

PGTDnoObs: cubePGTD ~ TestNo + PartnerEXP + JuvReun + PropPar + IOFLine + JuvVoc + PairAff + JuvReun * TestNo + PropPar * TestNo + IOFLine * TestNo + JuvVoc * TestNo + PairAff * TestNo + (1 | ID)

PGTDAll: cubePGTD ~ TestNo + ObsNo + PartnerEXP + JuvReun + PropPar + IOFLine + JuvVoc + PairAff + JuvReun * TestNo + PropPar * TestNo + IOFLine * TestNo + JuvVoc * TestNo + PairAff * TestNo + (1 | ID)

Null1: cubePGTD ~ 1 + (1 | ID)

Supplementary 6 Father Grate Touch Models:

FGTDonlyObsNo: cubeFGTD ~ ObsNo + (1 | ID)

FGTDonlyTestNo: cubeFGTD ~ TestNo + (1 | ID)

FGTDnoRel: cubeFGTD ~ TestNo + ObsNo + (1 | ID)

FGTDonlySig: cubeFGTD ~ TestNo + ObsNo + IOFProx + IOFVoc + IOFProx * TestNo + IOFVoc * TestNo + (1 | ID)

FGTDsigInt: cubeFGTD ~ TestNo + ObsNo + InfCarry + IOFProx + IOFVoc + JuvVoc + JuvLoc + ParAff + IOFProx * TestNo + IOFVoc * TestNo + ParAff * TestNo + (1 | ID)

FGTDnoPartEXP: cubeFGTD ~ TestNo + ObsNo + InfCarry + IOFProx + IOFVoc + JuvVoc + JuvLoc + ParAff + InfCarry * TestNo + IOFProx * TestNo + IOFVoc * TestNo + JuvVoc * TestNo + JuvLoc * TestNo + ParAff * TestNo + (1 | ID)

FGTDAll: cubeFGTD ~ TestNo + ObsNo + PartnerEXP + InfCarry + IOFProx + IOFVoc + JuvVoc + JuvLoc + ParAff + InfCarry * TestNo + IOFProx * TestNo + IOFVoc * TestNo + JuvVoc * TestNo + JuvLoc * TestNo + ParAff * TestNo + (1 | ID)

Null1: cubeFGTD ~ 1 + (1 | ID)

Supplementary 6 Social Zone Models:

PropSocialonlyTestNo: PropSocial ~ TestNo + (1 | ID)

PropSocialonlysig: PropSocial ~ TestNo + PropPar + JuvVoc + (1 | ID)

PropSocialnoInt: PropSocial ~ TestNo + PropPar + CnRPar + InfCarry + IOFGrate + IOFLine + IOFVoc + JuvVoc + (1 | ID)

PropSocialnoPartEXP: PropSocial ~ TestNo + PropPar + CnRPar + InfCarry + IOFGrate + IOFLine + IOFVoc + JuvVoc + PropPar * TestNo + CnRPar * TestNo + InfCarry * TestNo + IOFGrate * TestNo + IOFLine * TestNo + IOFVoc * TestNo + JuvVoc * TestNo + (1 | ID)

PropSocialnoObs: PropSocial ~ TestNo + PartnerEXP + PropPar + CnRPar + InfCarry + IOFGrate + IOFLine + IOFVoc + JuvVoc + PropPar * TestNo + CnRPar * TestNo + InfCarry * TestNo + IOFGrate * TestNo + IOFLine * TestNo + IOFVoc * TestNo + JuvVoc * TestNo + (1 | ID)

PropSocialAll: PropSocial ~ TestNo + ObsNo + PartnerEXP + PropPar + CnRPar + InfCarry + IOFGrate + IOFLine + IOFVoc + JuvVoc + PropPar * TestNo + CnRPar * TestNo + InfCarry * TestNo + IOFGrate * TestNo + IOFLine * TestNo + IOFVoc * TestNo + JuvVoc * TestNo + (1 | ID)

Null1: PropSocial ~ 1 + (1 | ID)

Supplementary 6 Other Zone Models:

ODonlyTestNo: sqrtOD ~ TestNo + (1 | ID)

ODonlysig: sqrtOD ~ TestNo + PropPar + JuvVoc + (1 | ID)

ODnoInt: sqrtOD ~ TestNo + PropPar + InfCarry + IOFGrate + IOFLine + IOFVoc + JuvVoc + JuvLoc + (1 | ID)

ODnoPartEXPorObsNo: sqrtOD ~ TestNo + PropPar + InfCarry + IOFGrate + IOFLine + IOFVoc + JuvVoc + JuvLoc + PropPar * TestNo + InfCarry * TestNo + IOFGrate * TestNo + IOFLine * TestNo + IOFVoc * TestNo + JuvVoc * TestNo + JuvLoc * TestNo + (1 | ID)

ODAll: sqrtOD ~ TestNo + ObsNo + PartnerEXP + PropPar + InfCarry + IOFGrate + IOFLine + IOFVoc + JuvVoc + JuvLoc + PropPar * TestNo + InfCarry * TestNo + IOFGrate * TestNo + IOFLine * TestNo + IOFVoc * TestNo + JuvVoc * TestNo + JuvLoc * TestNo + (1 | ID)

Null1: sqrtOD ~ 1 + (1 | ID)

Supplementary Table S7. Experiment 2 Log likelihood ratio test results comparing model fit for general linear mixed-effects models.

Supplementary 7. Social Salience Network

	npar	AIC	BIC	logLik	deviance	Chisq	Df	Pr(>Chisq)
ActivityonlyCondition	6	1554.26	1578.67	-771.13	1542.26			
ActivitynoRelation	11	211.96	256.71	-94.98	189.96	1352.297	5	< 2.2e-16
ActivitynoInt	13	202.4	255.29	-88.2	176.4	13.5593	2	0.001137
ActivitynoRegion	14	1556.99	1613.94	-764.49	1528.99	0	1	1
ActivitynoSide	19	62.16	139.46	-12.08	24.16	1504.824	5	< 2.2e-16
ActivityAll	20	63.89	145.26	-11.95	23.89	0.2668	1	0.605483
	npar	AIC	BIC	logLik	deviance	Chisq	Df	Pr(>Chisq)
Null1	3	1555.02	1567.22	-774.51	1549.02			
ActivitynoSide	19	62.16	139.46	-12.08	24.16	1524.9	16	< 2.2e-16

Supplementary 7. Periaqueductal Gray (PAG)

	npar	AIC	BIC	logLik	deviance	Chisq	Df	Pr(>Chisq)
ActivityonlyCondition	6	46.785	60.445	-17.392	34.785			
ActivityonlyJuvReun	7	40.998	56.934	-13.499	26.998	7.7871	1	0.005262
ActivitynoInt	12	36.005	63.325	-6.003	12.005	14.9922	5	0.010396
ActivityfourInt2	22	-9.24	40.846	26.62	-53.24	65.2455	10	3.64E-10
ActivityfourInt	24	-13.096	41.544	30.548	-61.096	7.8554	2	0.019689
ActivitynoSide	30	-8.48	59.82	34.24	-68.48	7.3841	6	0.286783
ActivityAll	31	-7.23	63.347	34.615	-69.23	0.7505	1	0.386323
	npar	AIC	BIC	logLik	deviance	Chisq	Df	Pr(>Chisq)
Null1	3	67.299	74.129	-30.65	61.299			
ActivityfourInt	24	-13.096	41.544	30.548	-61.096	122.39	21	2.59E-16

Supplementary 7. Cerebellum

	npar	AIC	BIC	logLik	deviance	Chisq	Df	Pr(>Chisq)
ActivityonlyCondition	6	32.9	42.401	-10.4499	20.8998			
ActivitynoParAff	7	27.025	38.11	-6.5126	13.0251	7.8747	1	0.005013
ActivitynoInt	8	25.285	37.953	-4.6427	9.2853	3.7398	1	0.053132
ActivityoneInt	11	20.667	38.086	0.6665	-1.333	10.6183	3	0.013979
ActivityAll	14	22.092	44.261	2.9541	-5.9082	4.5752	3	0.20568
	npar	AIC	BIC	logLik	deviance	Chisq	Df	Pr(>Chisq)
Null1	3	39.365	44.116	-16.6825	33.365			
ActivityoneInt	11	20.667	38.086	0.6665	-1.333	34.698	8	3.04E-05

Supplementary 7. Whole Brain

	npar	AIC	BIC	logLik	deviance	Chisq	Df	Pr(>Chisq)
ActivityonlyCondition	6	47.928	61.588	-17.9638	35.928			
ActivitynoCnR	9	33.986	54.476	-7.993	15.986	19.9416	3	0.0001745
ActivitynoInt	10	32.345	55.112	-6.1727	12.345	3.6405	1	0.0563884
ActivitytwoInt	16	10.858	47.284	10.5711	-21.142	33.4877	6	8.45E-06
ActivitynoSide	22	9.456	59.542	17.2721	-34.544	13.402	6	0.0370776
ActivityAll	23	11.264	63.628	17.3678	-34.736	0.1913	1	0.6618327
	npar	AIC	BIC	logLik	deviance	Chisq	Df	Pr(>Chisq)
Null1	3	68.361	75.191	-31.18	62.361			
ActivitynoSide	22	9.456	59.542	17.272	-34.544	96.905	19	1.94E-12

Supplementary 7. Cortisol (CORT)								
	npar	AIC	BIC	logLik	deviance	Chisq	Df	Pr(>Chisq)
CORTCondOnly	6	474.78	484.28	-231.39	462.78			
CORTnoPropPar	7	469.54	480.62	-227.77	455.54	7.2398	1	0.007131
CORTnoInt	8	467.22	479.88	-225.61	451.22	4.3224	1	0.037614
CORTonlyInf	10	467.29	483.12	-223.64	447.29	3.9286	2	0.140257
CORToneInt	11	464.96	482.38	-221.48	442.96	8.251	3	0.037614
CORTAll	14	468.85	491.02	-220.42	440.85	2.1178	3	0.548318
	npar	AIC	BIC	logLik	deviance	Chisq	Df	Pr(>Chisq)
Null1	3	474.52	479.27	-234.26	468.52			
CORToneInt	11	464.96	482.38	-221.48	442.96	25.556	8	0.001251

Supplementary 7 Social Salience Network Models:

ActivityonlyCondition: logTotalActivity ~ Condition + (1 | ID)

ActivitynoRelation: logTotalActivity ~ Condition + Region + (1 | ID)

ActivitynoInt: logTotalActivity ~ Condition + Region + JuvReun + ParAff + (1 | ID)

ActivitynoRegion: logTotalActivity ~ Condition + JuvReun + ParAff + JuvReun * Condition + ParAff * Condition + (1 | ID)

ActivitynoSide: logTotalActivity ~ Condition + Region + JuvReun + ParAff + JuvReun * Condition + ParAff * Condition + (1 | ID)

ActivityAll: logTotalActivity ~ Condition + Side + Region + JuvReun + ParAff + JuvReun * Condition + ParAff * Condition + (1 | ID)

Null1: logTotalActivity ~ 1 + (1 | ID)

Supplementary 7 Periaqueductal Gray (PAG) Models:

ActivityonlyCondition: logTotalActivity ~ Condition + (1 | ID)

ActivityonlyJuvReun: logTotalActivity ~ Condition + JuvReun + (1 | ID)

ActivitynoInt: logTotalActivity ~ Condition + JuvReun + CnRPar + IOFProx + IOFGrate + JuvVoc + ParAff + (1 | ID)

ActivityfourInt2: logTotalActivity ~ Condition + JuvReun + IOFGrate + JuvVoc + ParAff + JuvReun * Condition + IOFGrate * Condition + JuvVoc * Condition + ParAff * Condition + (1 | ID)

ActivityfourInt: logTotalActivity ~ Condition + JuvReun + CnRPar + IOFProx + IOFGrate + JuvVoc + ParAff + JuvReun * Condition + IOFGrate * Condition + JuvVoc * Condition + ParAff * Condition + (1 | ID)

ActivitynoSide: logTotalActivity ~ Condition + JuvReun + CnRPar + IOFProx + IOFGrate + JuvVoc + ParAff + JuvReun * Condition + CnRPar * Condition + IOFProx * Condition + IOFGrate * Condition + JuvVoc * Condition + ParAff * Condition + (1 | ID)

ActivityAll: logTotalActivity ~ Condition + Side + JuvReun + CnRPar + IOFProx + IOFGrate + JuvVoc + ParAff + JuvReun * Condition + CnRPar * Condition + IOFProx * Condition + IOFGrate * Condition + JuvVoc * Condition + ParAff * Condition + (1 | ID)

Null1: logTotalActivity ~ 1 + (1 | ID)

Supplementary 7 Cerebellum Models:

ActivityonlyCondition: logTotalActivity ~ Condition + (1 | ID)

ActivitynoParAff: logTotalActivity ~ Condition + JuvReun + (1 | ID)

ActivitynoInt: logTotalActivity ~ Condition + JuvReun + ParAff + (1 | ID)

ActivityoneInt: logTotalActivity ~ Condition + JuvReun + ParAff + JuvReun * Condition + (1 | ID)

ActivityAll: logTotalActivity ~ Condition + JuvReun + ParAff + JuvReun * Condition + ParAff * Condition + (1 | ID)

Supplementary 7 Whole Brain Models:

ActivityonlyCondition: logTotalActivity ~ Condition + (1 | ID)

ActivitynoCnR: logTotalActivity ~ Condition + JuvReun + IOFProx + IOFLine + (1 | ID)

ActivitynoInt: logTotalActivity ~ Condition + JuvReun + CnRPar + IOFProx + IOFLine + (1 | ID)

ActivitytwoInt: logTotalActivity ~ Condition + JuvReun + CnRPar + IOFProx + IOFLine + JuvReun * Condition + CnRPar * Condition + (1 | ID)

ActivitynoSide: logTotalActivity ~ Condition + JuvReun + CnRPar + IOFProx + IOFLine + JuvReun * Condition + CnRPar * Condition + IOFProx * Condition + IOFLine * Condition + (1 | ID)

ActivityAll: logTotalActivity ~ Condition + Side + JuvReun + CnRPar + IOFProx + IOFLine + JuvReun * Condition + CnRPar * Condition + IOFProx * Condition + IOFLine * Condition + (1 | ID)

Null1: logTotalActivity ~ 1 + (1 | ID)

Supplementary 7 Cortisol (CORT) Models:

CORTCondOnly: CORT ~ Condition + (1|ID)

CORTnoPropPar: CORT ~ Condition + InfCarry + (1|ID)

CORTnoInt: CORT ~ Condition + PropPar + InfCarry + (1|ID)

CORTonlyInf: CORT ~ Condition + InfCarry + InfCarry*Condition + (1|ID)

CORToneInt: CORT ~ Condition + PropPar + InfCarry + InfCarry*Condition + (1|ID)

CORTAll: CORT ~ Condition + PropPar + InfCarry + PropPar*Condition + InfCarry*Condition + (1|ID)

Null1: CORT ~ 1 + (1|ID)

SUPPLEMENTARY FIGURES

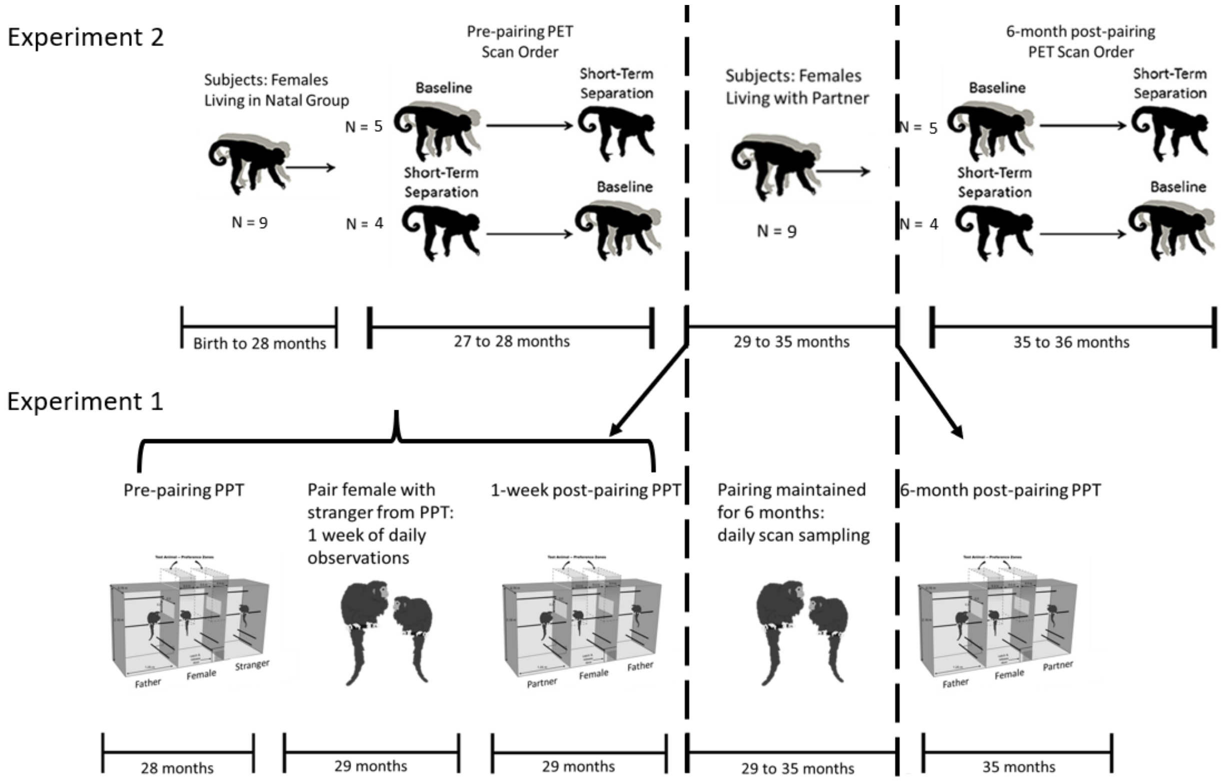

Supplementary Figure S1. Timeline for Experiments 1 and 2. Age of subject indicated in months. PET = Positron Emission Tomography; PPT = Partner Preference Test

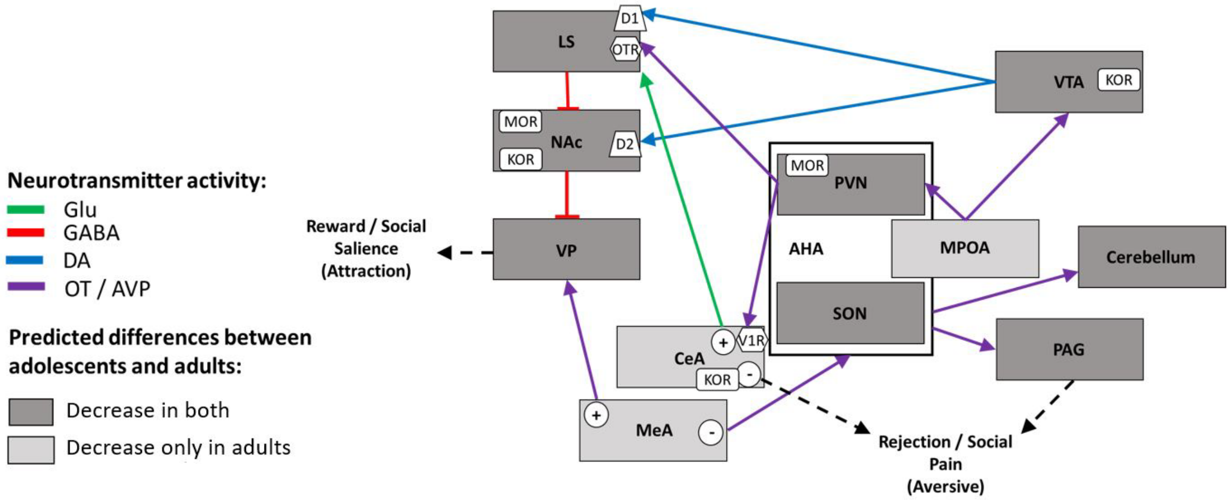

Supplementary Figure S2. Predictions for Experiment 2 regarding brain regions involved in father-daughter bonds and adult pair-bonds. Abbreviations: → = excitatory input; –| = inhibitory input; Glu = glutamate; GABA = Gamma aminobutyric acid; DA = dopamine; OT = oxytocin; AVP = arginine-vasopressin; D1 = dopamine receptor 1; D2 = dopamine receptor 2; OTR = oxytocin receptor; MOR = mu opioid receptor; KOR = kappa opioid receptor; V1R = arginine-vasopressin receptor 1; LS = lateral septum; NAc = nucleus accumbens; VP = ventral pallidum; CeA = central amygdala; MeA = medial amygdala; PVN = paraventricular nucleus of hypothalamus; SON = supraoptic nucleus of hypothalamus; AHA = anterior hypothalamus; MPOA = medial preoptic area of hypothalamus; VTA = ventral tegmental area; PAG = periaqueductal gray

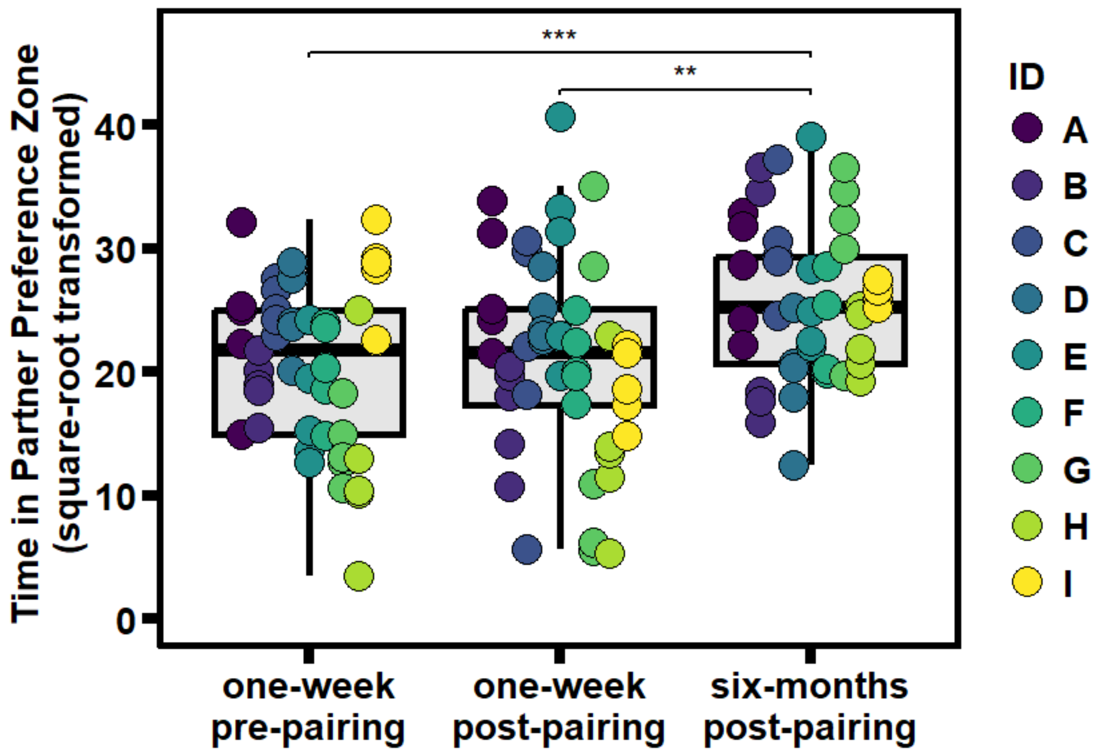

Supplementary Figure S3. Partner Zone duration: the main effect of *Test Number*. Females spent significantly more time in proximity to the partner during the six-months post-pairing test compared to both the one-week pre- and post-pairing tests. Points on the graph are colored based on subject identity. Significant differences for pairwise comparisons between tests indicated as: * < .05; ** < .01; *** < .001

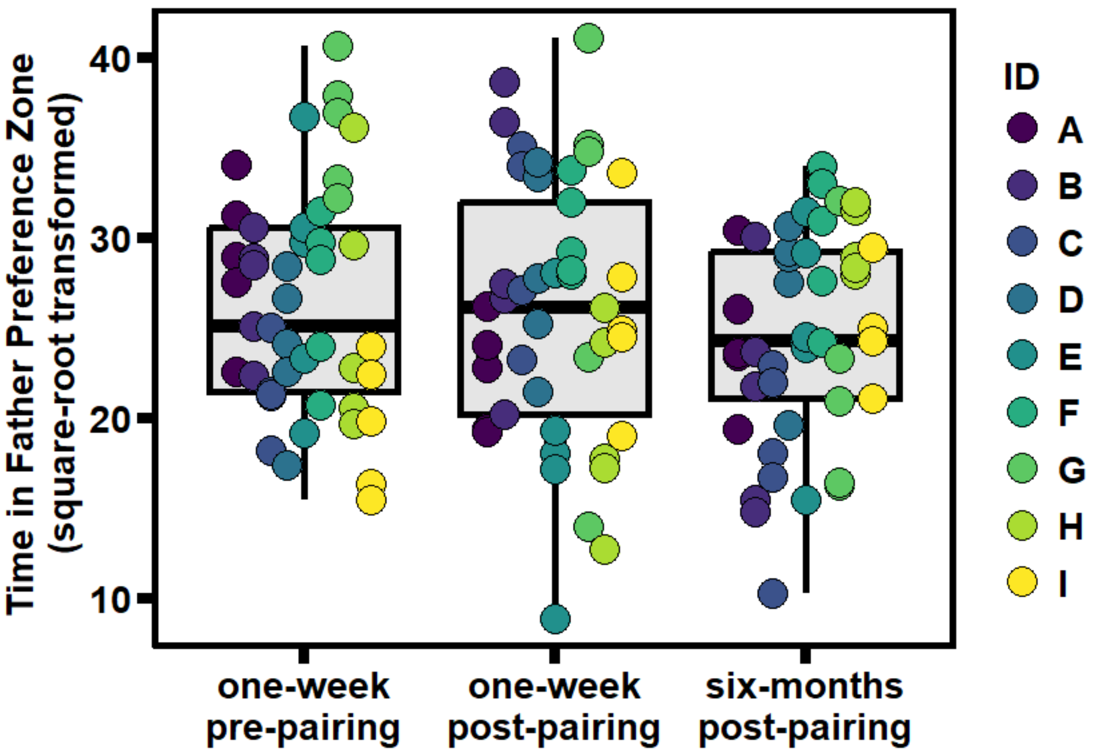

Supplementary Figure S4. Father Zone duration: the main effect of *Test Number.* Females did not significantly differ in the amount of time spent near the father across all three tests. Points on the graph are colored based on subject identity. Significant differences for pairwise comparisons between tests indicated as: * < .05; ** < .01; *** < .001

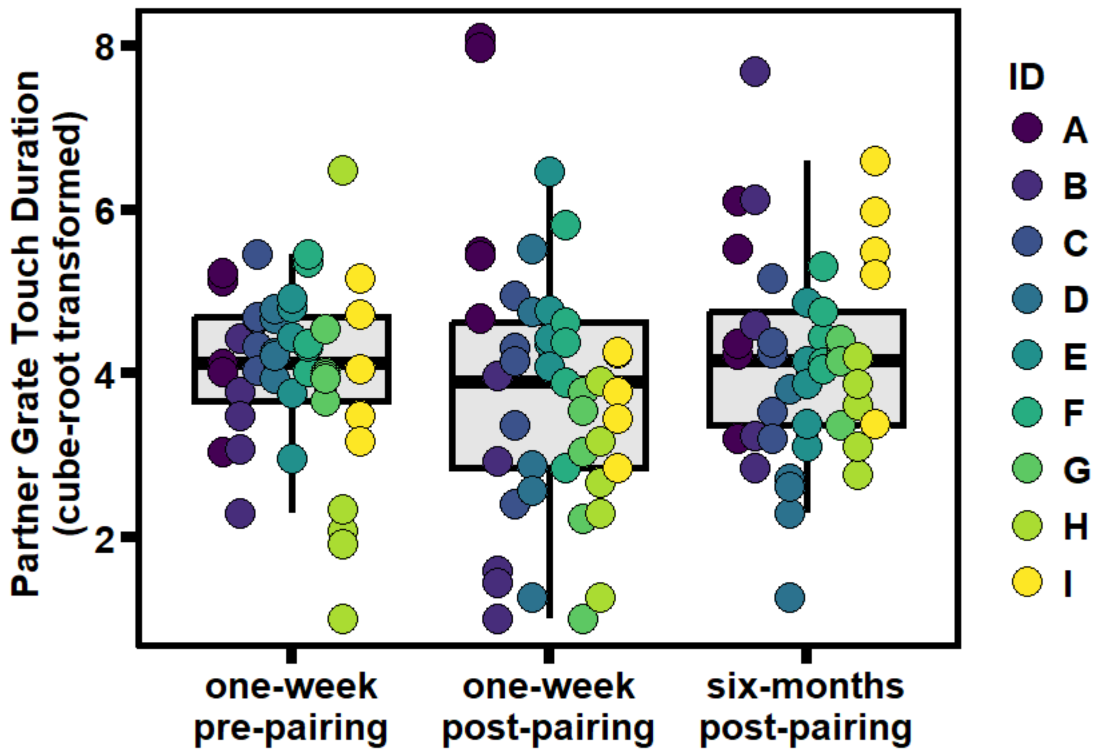

Supplementary Figure S5. Partner Grate Touch duration: the main effect of *Test Number.* Females did not significantly differ in the amount of time spent touching their partner's grate across all three tests. Points on the graph are colored based on subject identity. Significant differences for pairwise comparisons between tests indicated as: * < .05; ** < .01; *** < .001

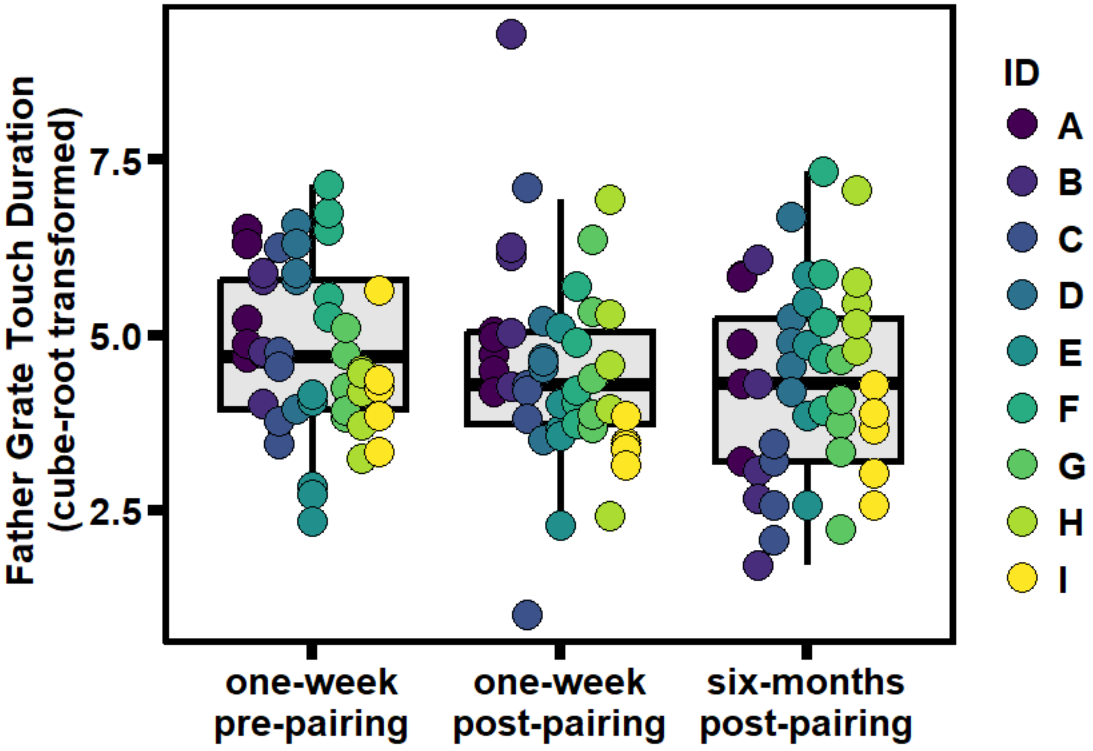

Supplementary Figure S6. Father Grate Touch duration: the main effect of *Test Number*. Females did not significantly differ in the amount of time spent touching their father's grate across all three tests. Points on the graph are colored based on subject identity. Significant differences for pairwise comparisons between tests indicated as: * < .05; ** < .01; *** < .001

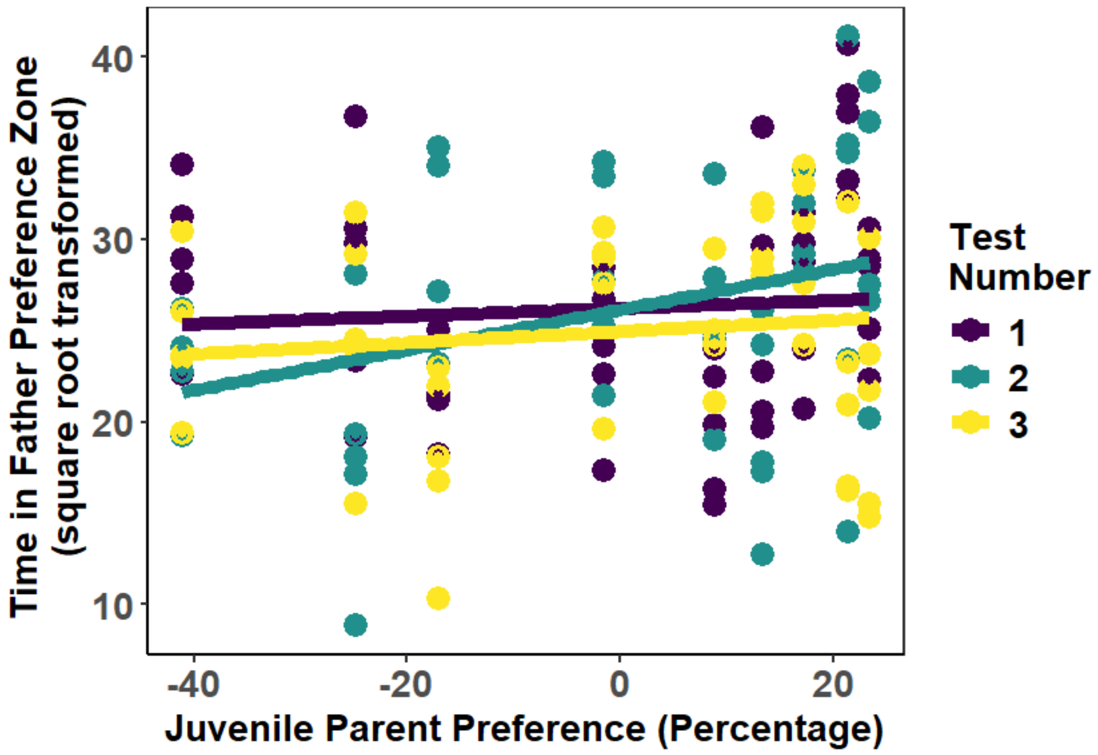

Supplementary Figure S7. Father Zone duration: a) the interaction effect between *Test Number* and *Juvenile Parent Preference.* There was a non-significant trend towards females spending more time in proximity to their fathers if they also spent more time in proximity to their parents during juvenile preference testing. Test Number 1 = 1-week pre-pairing; Test Number 2 = 1-week post-pairing; Test Number 3 = 6-months post-pairing.

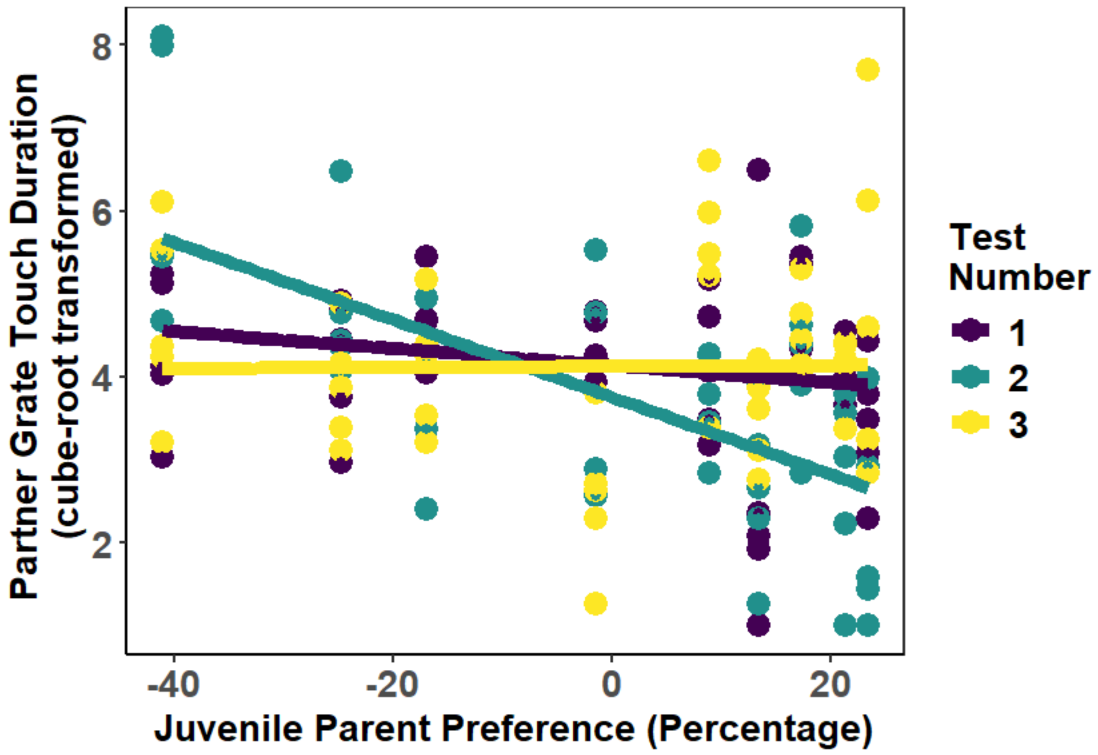

Supplementary Figure S8. Partner Grate Touching duration: a) the interaction effect between *Test Number* and *Juvenile Parent Preference.* Females that spent a greater amount of time in their parents' preference zone during juvenile testing spent less time touching the partner's grate. Test Number 1 = 1-week pre-pairing; Test Number 2 = 1-week post-pairing; Test Number 3 = 6-months post-pairing.

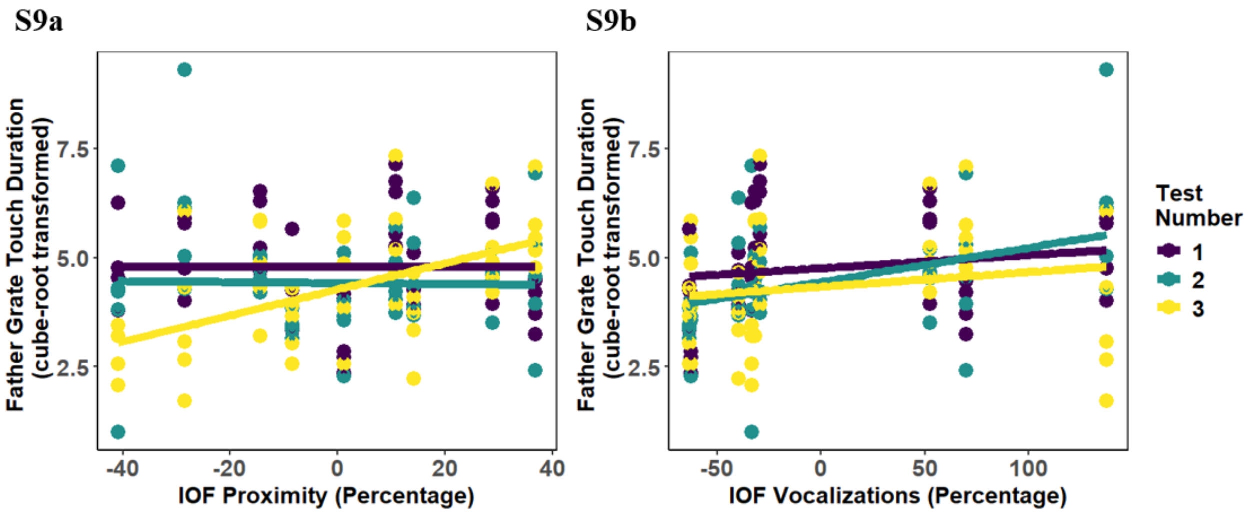

Supplementary Figure S9. Father Grate Touching duration: a) the interaction effect between *Test Number* and a) *IOF Proximity* and b) *IOF Vocalizations.* Females that exhibit a greater increase in time in proximity to their father during IOF testing (*IOF Proximity*) spend more time touching the father's grate. Females that exhibit a greater increase in vocalizations when separated from their father during IOF testing (*IOF Vocalizations*) spend more time touching their father's grate during test two. Test Number 1 = 1-week pre-pairing; Test Number 2 = 1-week post-pairing; Test Number 3 = 6-months post-pairing.

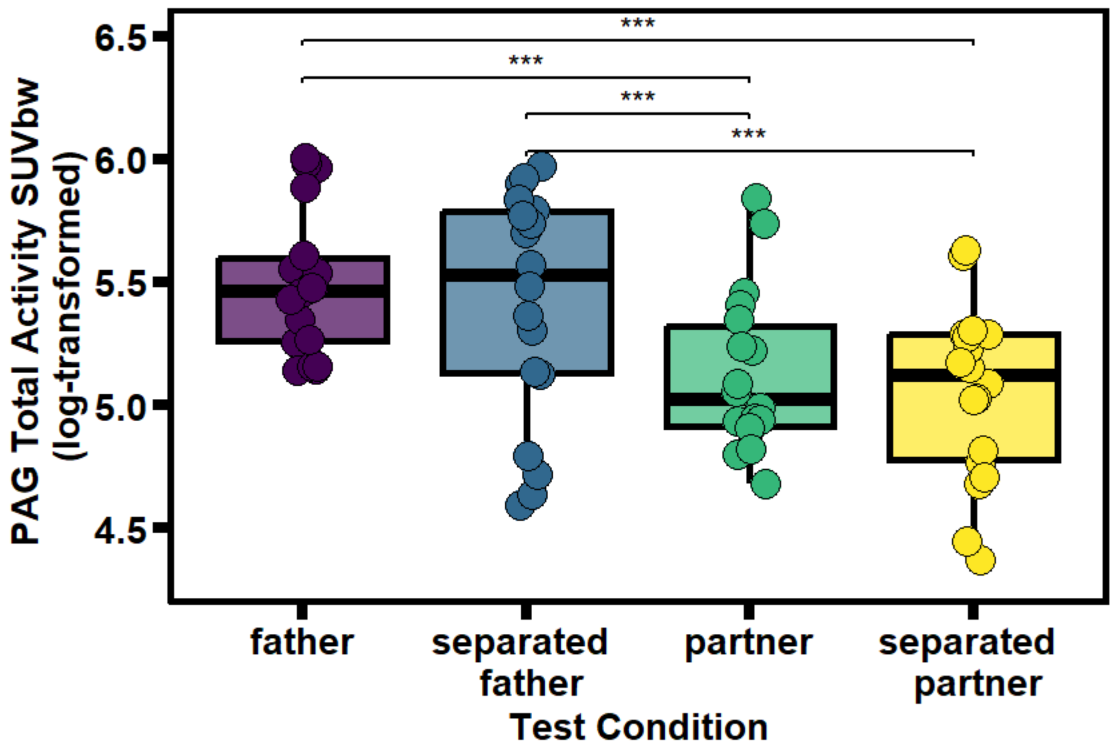

Supplementary Figure S10. Periaqueductal Gray glucose uptake (SUVbw): the main effect of *Condition.* Periaqueductal gray activity was lower when females were tested with their partner and when they were separated from their partner compared to when they were tested with their father while in their natal group. father = when tested with father when still in natal group; partner = when tested with partner 6-months post-pairing; separated father = when separated from father while still in natal group; separated partner = when separated from partner 6-months post-pairing; SUVbw = Total Activity (glucose uptake) calculated as Standardized Uptake Value by body weight; PAG = Periaqueductal Gray. Significant differences for pairwise comparisons between tests indicated as: * < .05; ** < .01; *** < .001

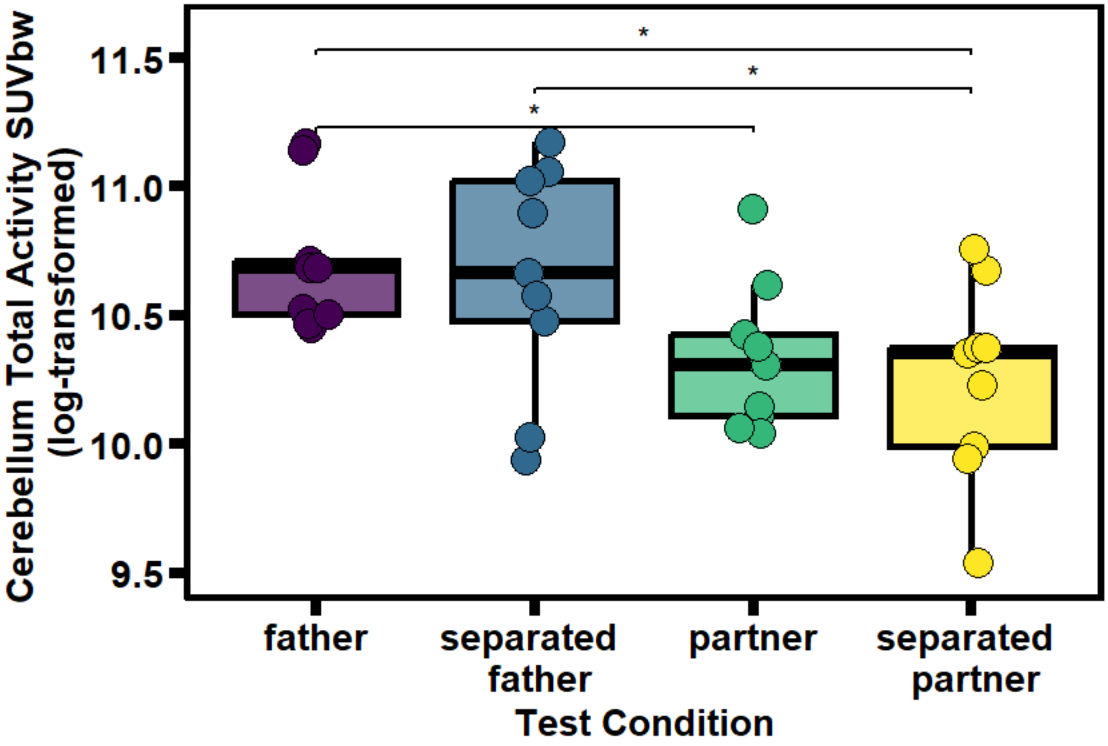

Supplementary Figure S11. Cerebellum glucose uptake (SUVbw): the main effect of *Condition*. Cerebellum activity was lower when females were tested with their partner and when they were separated from their partner compared to when they were tested with their father while in their natal group. father = when tested with father when still in natal group; partner = when tested with partner 6-months post-pairing; separated father = when separated from father while still in natal group; separated partner = when separated from partner 6-months post-pairing; SUVbw = Total Activity (glucose uptake) calculated as Standardized Uptake Value by body weight. Significant differences for pairwise comparisons between tests indicated as: * < .05; ** < .01; *** < .001

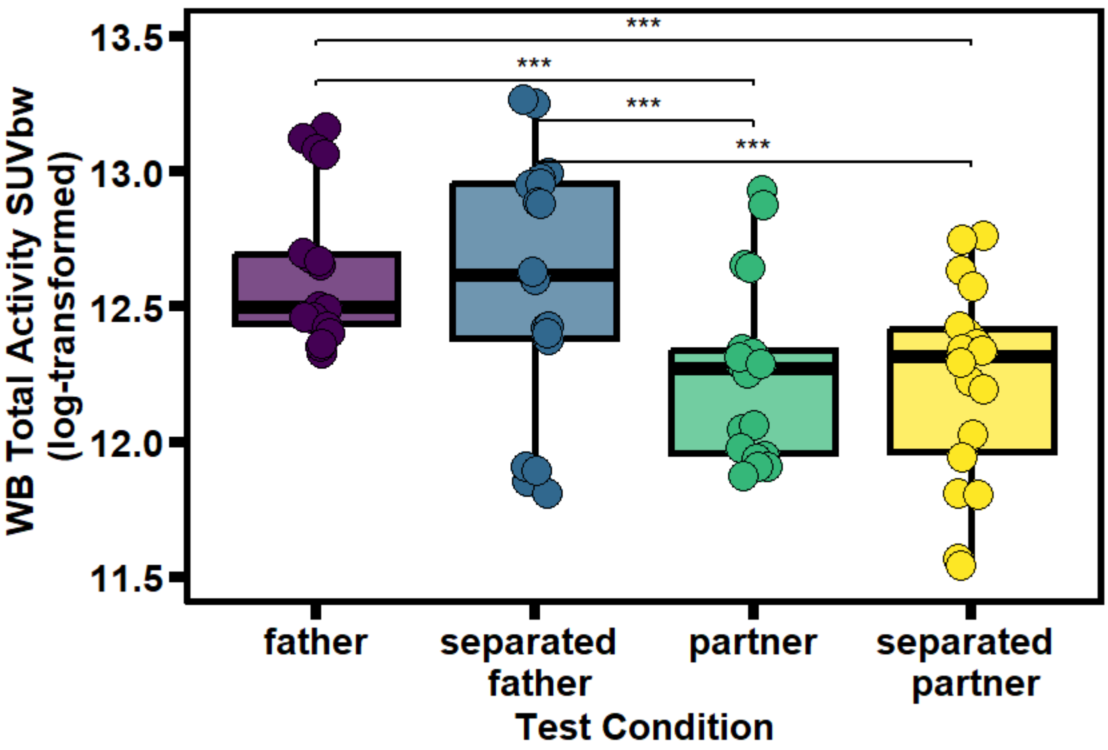

Supplementary Figure S12. Whole Brain glucose uptake (SUVbw): the main effect of *Condition.* Whole brain activity was lower when females were tested with their partner and when they were separated from their partner compared to when they were tested with their father while in their natal group. father = when tested with father when still in natal group; partner = when tested with partner 6-months post-pairing; separated father = when separated from father while still in natal group; separated partner = when separated from partner 6-months post-pairing; SUVbw = Total Activity (glucose uptake) calculated as Standardized Uptake Value by body weight; WB = Whole Brain. Significant differences for pairwise comparisons between tests indicated as: * < .05; ** < .01; *** < .001

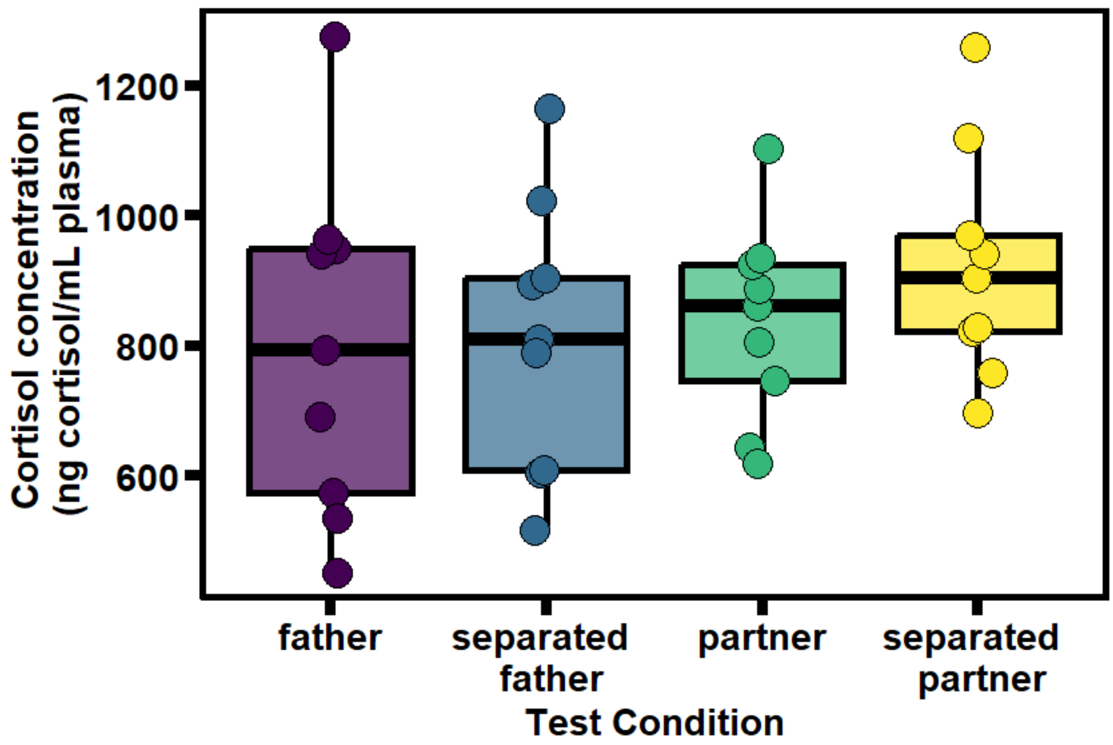

Supplementary Figure S13. Cortisol: the main effect of *Condition.* Plasma cortisol taken immediately following the 30-minute test conditions did not significantly differ between any of the four tests (stress buffered with attachment figure or separation distress separated from attachment figure pre- and post-pairing). father = when tested with father when still in natal group; partner = when tested with partner 6-months post-pairing; separated father = when separated from father while still in natal group; separated partner = when separated from partner 6-months post-pairing. Significant differences for pairwise comparisons between tests indicated as: * < .05; ** < .01; *** < .001

S14a S14b

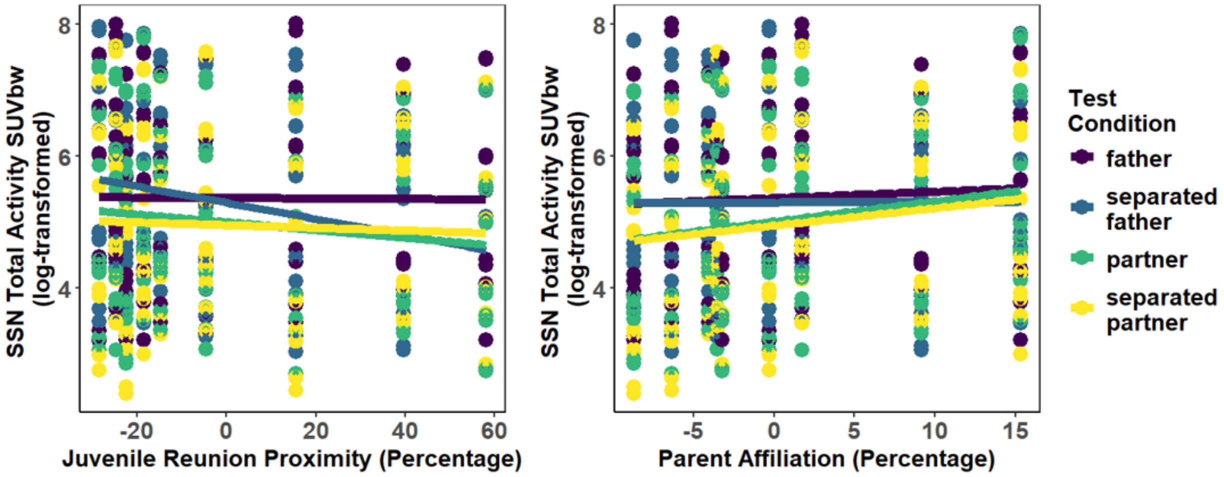

Supplementary Figure S14. Social Salience Network glucose uptake (SUVbw): the interaction effect between *Condition* and a) *Juvenile Proximity* and b) *Parent Affiliation. Juvenile Proximity* is negatively related to Total Activity when females are tested with their partner and separated from their father, suggesting females that spend a greater percentage of time in proximity to their fathers following a separation exhibit lower activity of the social salience network during these two test conditions. We found a positive relation between *Parent Affiliation* and Total Activity when females were with their partners and separated from their partners, suggesting they exhibited higher activity of the social salience network during these two conditions if their parents spent a greater percentage of time in affiliative contact when they were juveniles. father = when tested with father when still in natal group; partner = when tested with partner 6-months post-pairing; separated father = when separated from father while still in natal group; separated partner = when separated from partner 6-months post-pairing; SUVbw = Total Activity (glucose uptake) calculated as Standardized Uptake Value by body weight; SSN = Social Salience Network

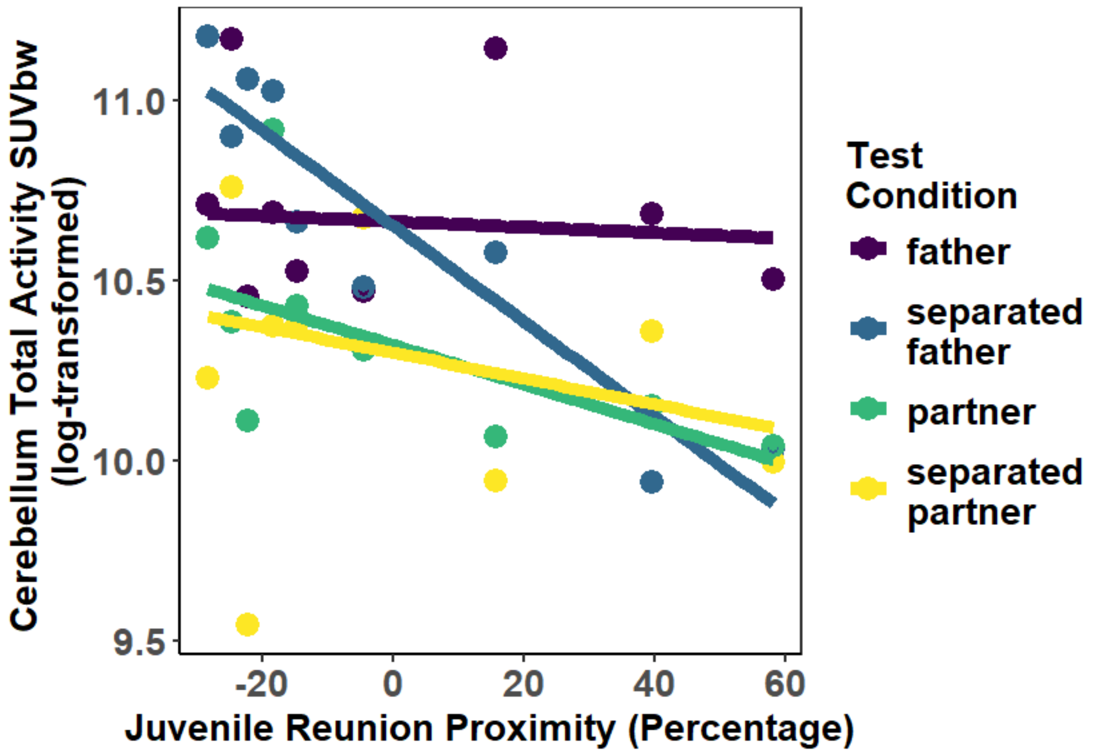

Supplementary Figure S15. Cerebellum glucose uptake (SUVbw): the interaction effect between *Condition* and *Juvenile Proximity.* We found a negative relation between *Juvenile Proximity* and Total Activity in the cerebellum when females were tested separated from their fathers, suggesting females that spend a greater amount of time in proximity to their fathers following juvenile separation testing exhibit lower cerebellum activity when separated from their father. father = when tested with father when still in natal group; partner = when tested with partner 6-months post-pairing; separated father = when separated from father while still in natal group; separated partner = when separated from partner 6-months post-pairing; SUVbw = Total Activity (glucose uptake) calculated as Standardized Uptake Value by body weight

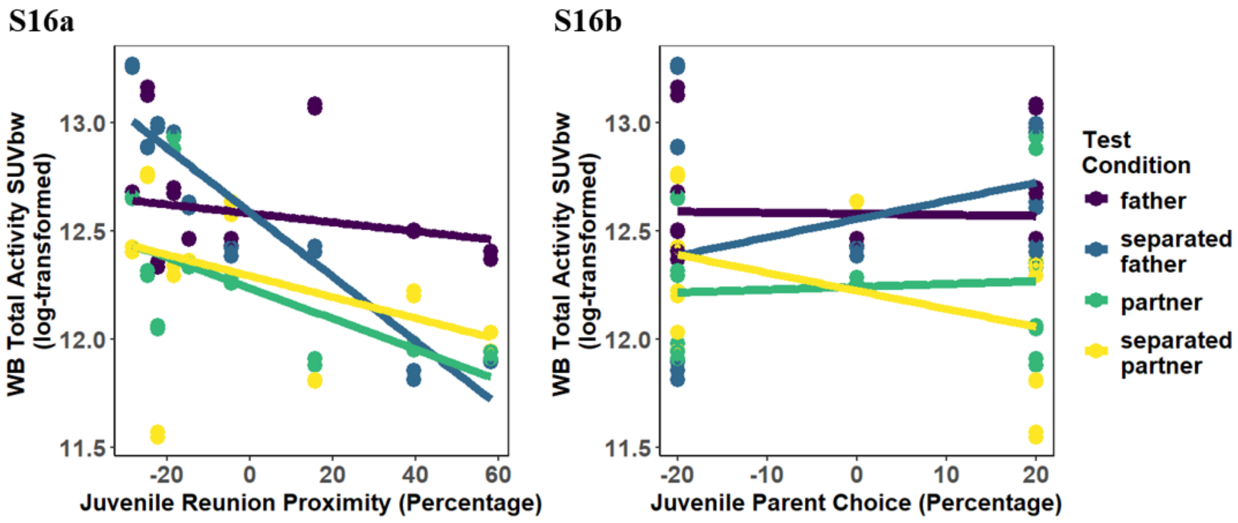

Supplementary Figure S16. Whole Brain glucose uptake (SUVbw): the interaction effect between *Condition* and a) *Juvenile Proximity* and b) *Juvenile Parent Choice.* We found a negative relation between *Juvenile Proximity* and Total Activity when females were tested with their partners and separated from their fathers, suggesting females that spend a greater amount of time in proximity to their fathers following juvenile separation testing exhibit lower whole brain activity in these two conditions. We found a negative relation between *Juvenile Parent Choice* and Total Activity when females were separated from their partners, suggesting females that choose their parents a greater percentage of time during catch and release testing have lower whole brain activity when separated from their partners. father = when tested with father when still in natal group; partner = when tested with partner 6-months post-pairing; separated father = when separated from father while still in natal group; separated partner = when separated from partner 6-months post-pairing; SUVbw = Total Activity (glucose uptake) calculated as Standardized Uptake Value by body weight; WB = Whole Brain

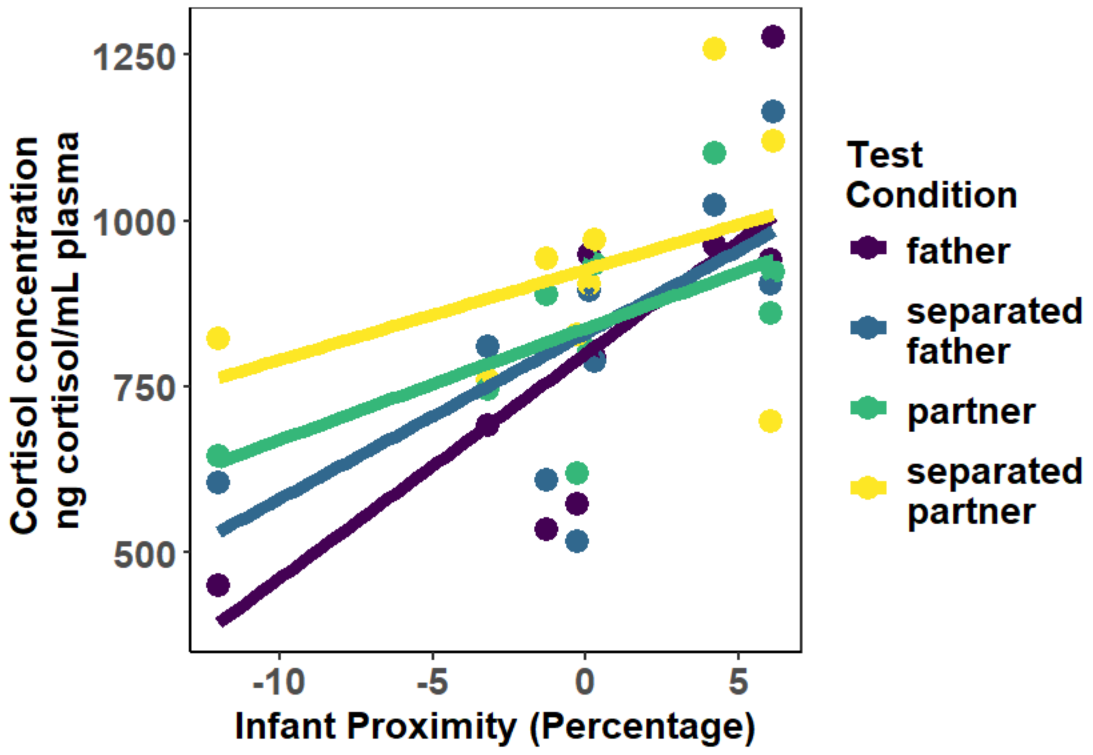

Supplementary Figure S17. Cortisol concentration (ng cortisol/mL plasma): the interaction effect between *Condition* and *Infant Proximity*. Females that spent more time in proximity to their fathers over the first nine months of their lives had a significantly higher cortisol concentration when separated from their partner.

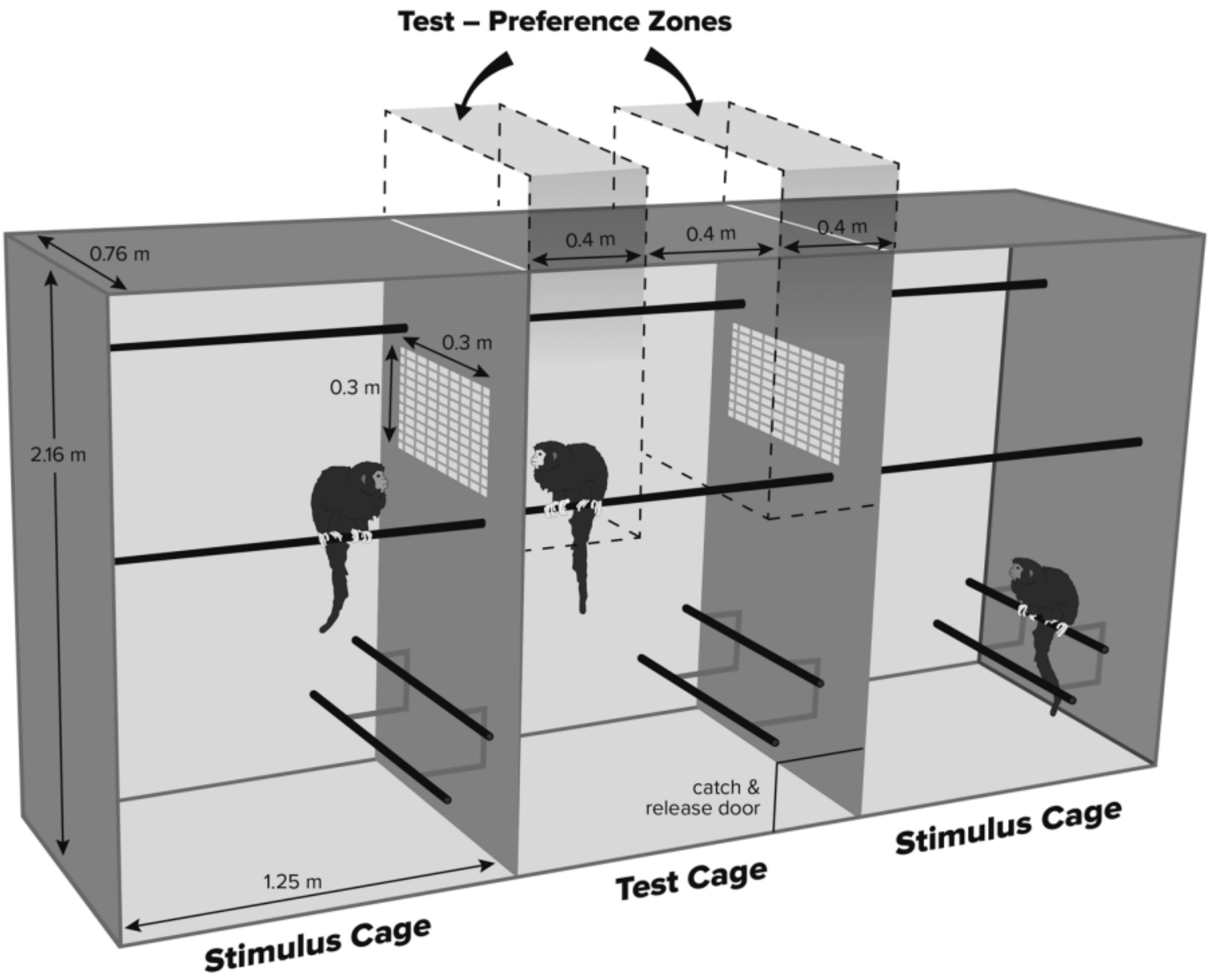

Supplementary Figure S18. Schematic of Experiment 1 preference test arena (adapted from Rothwell et al., 2020).

S19a S19b

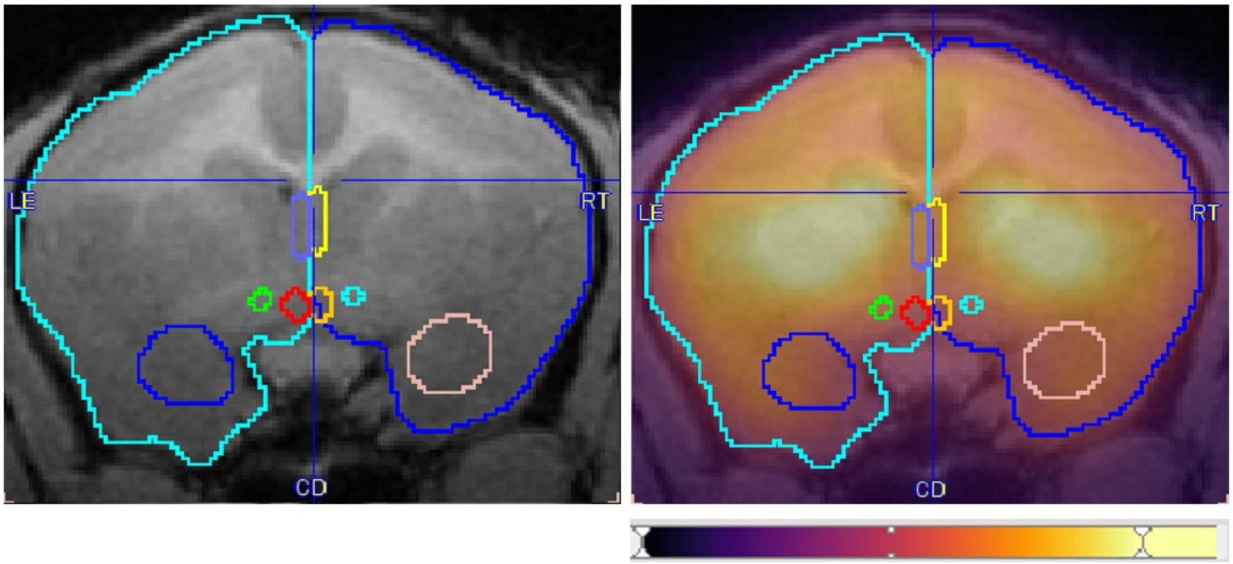

Supplementary Figure S19. Example segmentation of regions of interest (ROIs). a) Magnetic resonance image (MRI) with segmentation of hypothalamus (left = orange; right = red), ventral pallidum (left = light blue; right = green), lateral septum (left = yellow; right = purple), and amygdala (left = pink; right = dark blue), left whole brain (dark blue outline around left hemisphere), and right whole brain (light blue outline around right hemisphere). b) Positron Emission Tomography (PET) image co-registered with MRI, including segmented ROIs. Warmer colors (e.g., yellow) indicate higher glucose uptake (see scale below co-registered image).
